# Emotional reactions to climate change: a comparison across France, Germany, Norway, and the United Kingdom

**DOI:** 10.3389/fpsyg.2023.1139133

**Published:** 2023-07-06

**Authors:** Gisela Böhm, Hans-Rüdiger Pfister, Rouven Doran, Charles A. Ogunbode, Wouter Poortinga, Endre Tvinnereim, Katharine Steentjes, Claire Mays, Raquel Bertoldo, Marco Sonnberger, Nicholas Pidgeon

**Affiliations:** ^1^Department of Psychosocial Science, Faculty of Psychology, University of Bergen, Bergen, Norway; ^2^Department of Psychology, Inland Norway University of Applied Sciences, Lillehammer, Norway; ^3^Institute of Experimental Industrial Psychology (LueneLab), Leuphana University Lüneburg, Lüneburg, Germany; ^4^School of Psychology, University of Nottingham, Nottingham, United Kingdom; ^5^Welsh School of Architecture, Cardiff University, Cardiff, United Kingdom; ^6^Centre for Climate Change and Social Transformations (CAST), School of Psychology, Cardiff University, Cardiff, United Kingdom; ^7^Department of Government, Faculty of Social Sciences, University of Bergen, Bergen, Norway; ^8^NORCE Norwegian Research Centre, Bergen, Norway; ^9^Institut Symlog, Paris, France; ^10^Aix Marseille Univ, LPS, Aix-en-Provence, France; ^11^Department of Sociology of Technology, Risk and Environment, University of Stuttgart, Stuttgart, Germany; ^12^Section for Environmental Sociology, Friedrich-Schiller-University Jena, Jena, Germany; ^13^Understanding Risk Research Group, School of Psychology, Cardiff University, Cardiff, United Kingdom

**Keywords:** climate change, emotions, appraisal theories, risk perception, sustainability, environmental behavior, cross-national comparison

## Abstract

We present a study of emotional reactions to climate change utilizing representative samples from France, Germany, Norway, and the United Kingdom (UK). Drawing on appraisal theories of emotion, we examine relations between appraisals, emotions, and behavioral intentions in the context of climate change. We compare the four countries concerning emotional differences and commonalities and relate our findings to pertinent models of cultural values. Five distinct emotions were measured: worry, hope, fear, outrage, and guilt. In addition, the survey asked respondents to appraise a set of climate-related statements, such as the causality of climate change, or the efficacy of mitigation efforts. Also, a set of climate-relevant actions, such as willingness to reduce energy consumption or support for climate policies, was assessed. Findings show that appraisals of human causation and moral concern were associated with worry and outrage, and appraisals of efficacy and technological solutions were associated with hope. Worry and outrage are associated with intentions to reduce one’s energy consumption, whereas hope and guilt are related to support for policies such as tax and price increases. A country comparison shows that French respondents score high on outrage and worry and tend to engage in individual behaviors to mitigate climate change, whereas Norwegian respondents score high on hope and show a tendency to support policies of cost increase. Generally, worry is the most and guilt the least intense emotion. Moral concerns and perceived collective efficacy of one’s country in addressing climate change are relatively strong in France, while beliefs in human causation and in negative impacts of climate change prevail in Germany, and confidence in technological solutions are prevalent in Norway. In sum, findings reveal typical patterns of emotional responses in the four countries and confirm systematic associations between emotions and appraisals as well as between emotions and behaviors. Relating these findings to models of cultural values reveals that Norway, endorsing secular and egalitarian values, is characterized by hope and confidence in technological solutions, whereas France and Germany, emphasizing relatively more hierarchical and traditional values, are rather characterized by fear, outrage, and support for behavioral restrictions imposed by climate change policies.

## 1. Introduction

Emotions have been shown to be a core part of any kind of individual or collective response to climate change (Davidson and Kecinski, 2022). In this study, we examine five discrete emotions (worry, fear, hope, outrage, and guilt) that may be most prototypical in relation to climate change (Böhm and Pfister, 2000, 2017; Böhm, 2003). We analyze the prevalence of these five climate-relevant emotions, and how they relate to both cognitive appraisals of climate change and climate behaviors. We also explore differences between countries concerning people’s emotional experiences in the context of climate change. Our first research question, thus, is concerned with the general relationship between emotions, appraisals, and behaviors in the context of climate change. The second research question focuses on national differences concerning emotional responses to climate change.

The study uses survey data that were collected concurrently in four European countries: France, Germany, Norway, and the United Kingdom. These countries represent four key energy producing nations in Europe, and vary in significant ways with respect to energy production, vulnerability to the impacts of climate change, and background socio-political contexts (Arnold et al., 2016; Steentjes et al., 2017). Their comparison promises to help inform future research on which socio-economic or cultural factors may be worthwhile exploring in more depth with respect to their role in climate emotions, underlying appraisals, and subsequent climate behaviors.

To our knowledge, this is the first study that investigates several discrete emotions in an international comparison of representative samples of the adult population and which considers a broader range of both antecedents and consequences of emotions. The results can inform future studies and contribute to our understanding of how emotions shape judgments and behaviors related to sustainability issues such as climate change. The results may further be beneficially used to inform emotion-based communications that aim at fostering public climate engagement.

### 1.1. Types of climate-relevant emotions, appraisals, and behaviors

There is broad consensus in the literature that emotions “*are internal states that arise following appraisals (evaluations) of interpersonal or intrapersonal events that are relevant to an individual’s concerns … and promote certain patterns of response*” (Cowen et al., 2019, p. 72f). Affect and emotions are recognized in the literature as an integral part of judgment and decision making (Peters et al., 2006; Böhm and Brun, 2008; Pfister and Böhm, 2008, 2017). In particular, the role of affect or emotions in shaping climate-related perceptions and judgments as well as sustainable behaviors is becoming increasingly clear (for recent reviews, see Brosch, 2021, and Davidson and Kecinski, 2022). Affect or emotions have been found to be among the strongest predictors of climate-related risk perceptions (van der Linden, 2015), mitigation (Xie et al., 2019) and adaptation (van Valkengoed and Steg, 2019) behaviors, and preferences for energy technologies (Jobin and Siegrist, 2018; Sonnberger et al., 2021).

Many, if not most, studies investigating the role of emotions in climate-related perceptions, judgments and behaviors have done so in terms of general positive or negative affect, with a greater focus on the latter (van der Linden, 2015; Jobin and Siegrist, 2018; Xie et al., 2019). However, it has long been known that different discrete emotions sharing the same valence can have distinct impacts on judgments and behavior (Böhm and Pfister, 2000; Pfister and Böhm, 2008, 2017). For example, it has been shown that fear increases risk perception, while anger attenuates it (Lerner and Keltner, 2000).

This is in line with appraisal theories of emotion, which we will follow in our approach. Appraisal theories hold that discrete emotions result from specific cognitive appraisals and motivate specific behavioral responses (Frijda et al., 1989). From this perspective, emotions experienced in relation to climate change are a complex response that reflects both how people interpret the phenomenon and how they respond to it (Chapman et al., 2017; Brosch, 2021). Appraisal theories can therefore explain how different discrete emotions that are of the same valence can have different effects on judgments and behaviors. For example, a person reacting with fear to climate change is likely to anticipate severe harmful impacts and to engage in mitigative or adaptive actions, whereas an angry person focuses more on assigning blame to a perpetrator and is likely to show a preference for punitive actions (Böhm and Pfister, 2000, 2017).

There is a great variety and complexity of emotional experiences, and their role in judgment and behavior is similarly diverse (Pfister and Böhm, 2008, 2017). There is no fixed set of emotions that can be said to cover all emotional experiences within the context of environmental issues in general and climate change in particular. A handful of taxonomies of climate- or eco-related emotions have been formulated, most recently by Pihkala (2022). The Pihkala (2022) taxonomy of climate emotions is based on a comprehensive review of the literature and consists of the broad categories of surprise, shock, fear, sadness, strong depression, guilt, feeling betrayed, anger, hostility, envy, motivation, pleasure, hope, belonging, and compassion, reflecting groups of similar types of emotions.

Landmann (2020) has taken a more theory-driven approach in the context of environmental protection, which builds on three previous classifications of emotions (Böhm, 2003; Kals and Müller, 2012; Hahnel and Brosch, 2018). Landmann (2020) distinguishes seven distinct types of emotions: self-condemning emotions (e.g., guilt, shame, embarrassment); other-condemning emotions (e.g., anger, disgust, contempt); self-praising emotions (e.g., pride); other-praising emotions (e.g., admiration, gratitude); other-suffering emotions (e.g., compassion); threat-related emotions (e.g., fear); and hedonistic emotions (e.g., pleasure). Two emotion types not included in the Landmann (2020) typology, but which may reflect relevant appraisals in the context of climate change, are loss-based emotions (e.g., sadness), and emotions based on positive future prospects (e.g., hope) (Böhm, 2003).

Brosch’s (2021) review of the literature discusses the role of emotions in climate perceptions and judgments as well as in driving climate action (see also Landmann, 2020; Pihkala, 2022). According to Brosch (2021), worry, hope, and fear are all future-oriented and imply that a person focuses on potential future consequences (Böhm and Pfister, 2000; Böhm, 2003). However, while hope implies that a negative outcome is avoidable or a positive outcome achievable, worry and fear anticipate exclusively negative outcomes. Worry is arguably the most frequently studied climate-related emotion (van der Linden, 2017; Bouman et al., 2020; Gregersen et al., 2020). Worry is closely related to perceived risk, reflecting an emotional reaction to uncertain harmful future outcomes (Borkovec et al., 1983). The behavioral response to worry is likely to be aimed at reducing a particular threat (van der Linden, 2017). Worry has indeed been found to be a reliable predictor of various forms of climate engagement, such as personal mitigating behaviors (Bouman et al., 2020; Gregersen et al., 2021) and policy support (Smith and Leiserowitz, 2014; Bouman et al., 2020).

Fear is akin to worry in that it is also a threat-related response, but it is more intense, short-lived, and directed toward an immediate threat (Borkovec et al., 1983; Ojala et al., 2021). Fear is considered a strong motivator of preventive action, as is illustrated by the fact that appealing to fear is a common strategy to promote behavioral change, especially in the health domain. The general finding is that strong fear appeals can be effective but need to be combined with high-efficacy messaging (inducing the perceived ability to achieve something or to attain a given goal); otherwise, they may produce defensive responses such as denial and avoidance (Witte and Allen, 2000). Some authors have argued that communicating efficacy may be more important for promoting protective action than fear appeals in themselves (Ruiter et al., 2014). In the context of climate change, empirical evidence on the effectiveness of fear appeals is sparse (Reser and Bradley, 2017), but it can be expected that their effectiveness depends on whether behavioral options are available that are easy to perform and perceived as effective in addressing climate change (Bostrom et al., 2018).

It has been argued that positive emotions need greater attention in relation to climate change (Stern, 2012; Schneider et al., 2021), especially because fear appeals have the potential to be counterproductive (Bostrom et al., 2018). While positive emotions have generally been studied less than negative emotions in the context of environment and climate change, hope has received some attention within the literature (Pihkala, 2022). Hope reflects optimism and empowerment (Pihkala, 2022). Hope has been associated with support for climate change policies in one of the earliest studies on climate emotions (Smith and Leiserowitz, 2014). Hope may, however, also lead to complacency rather than action (Hornsey and Fielding, 2020). For hope to promote climate action, it seems necessary that the underlying appraisals focus on the importance of acting (Brosch, 2021).

Climate change is considered a moral issue by many (Bassarak et al., 2017). Issues of social justice are raised due to the social, geographical, and temporal distance between causers and victims of climate change (Pawlik, 1991). Moral emotions, such as guilt, shame, and outrage, are therefore important to consider when discussing climate emotions (Böhm and Pfister, 2000; Böhm, 2003; Kals and Müller, 2012). Böhm and Pfister (Böhm and Pfister, 2000, 2005, 2017; Böhm, 2003) proposed and tested an appraisal-theoretical model that explains the antecedents and consequences of moral emotions in relation to moral risks. They show that moral emotions are based on moral judgments such as perceived moral blameworthiness (Böhm and Pfister, 2000, 2005, 2017). These moral emotions are triggered when an environmental risk is attributed to human causation and intensify with the ease with which responsibility can be ascribed to an identifiable perpetrator (Böhm and Pfister, 2000).

Self-related moral emotions, such as guilt, are predicted by judgments of individual contribution to the problem (Böhm, 2003). Other authors have reported similar findings. Ferguson and Branscombe (2010) show that collective guilt for carbon emissions depends on causal beliefs about global warming, such as belief in human causation, and promotes willingness to engage in mitigation behaviors. In work conducted by Harth et al. (2013), responsibility for environmental damage of one’s in-group increased both guilt and anger. Whereas guilt predicted intentions to contribute to repairing the damage, anger was related to support for punishing environmental sinners.

In sum, worry, fear, outrage, hope, and guilt can be viewed as a core set of emotions relevant in the context of climate change. As argued above, these five emotions are plausible candidates to encompass the diversity of emotional experiences of climate change in a parsimonious manner and will be used as primary variables in our study.

### 1.2. Contextual effects of emotional experiences

There are not only psychological but also sociological roots of emotions (Davidson and Kecinski, 2022). Emotions are social constructions since they depend on an individual’s interpretation of situations, events, or other phenomena which trigger specific emotions. These interpretations are always shared among certain groups of people, and thus are cultural phenomena, since they are the basis of meaningful interaction between individuals (Gerhards, 1989; Davidson and Kecinski, 2022). Furthermore, as Hochschild (1979) has pointed out, emotions are also shaped by feeling rules that determine what appropriate feelings are. As cultural norms, these feeling rules are shared among specific groups of people. Since countries are relevant boundaries of cultural norms, it can be assumed that feeling rules and thus dominant emotions associated with climate change vary across countries. In sum, emotions are as much cultural as psychological products. Culture as collective construction of meaning not only consists of shared and dominant value orientations but also of corresponding types of emotions (Gerhards, 1989). Value orientations and types of emotions are therefore likely to vary between countries. Furthermore, research has shown that socio-economic structures in the form of material conditions influence dominant emotions (Manstead, 2018).

Climate-related appraisals are part of socially constructed meaning (Krimsky and Golding, 1992; Slovic, 1999), and can be socially amplified through the public debate, especially if there is substantial controversy about the issue (as in the case of climate change; Renn, 2011). Distinct interpretative communities have been identified that each respond to climate change in their own way. A popular approach in this regard is the work by Leiserowitz et al. (2023) who distinguish Global Warming’s Six Americas, a segmentation of the United States American population in six distinct communities ranging from the Alarmed to the Dismissive and differing in how strongly their members believe in the existence and the human causation of climate change, how serious a threat they perceive climate change to be, and how much they support climate policies. These communities have been found to be linked to demographic variables: younger generations, people of color, and women are more likely to be found at the alarmed end of the spectrum and less likely to be dismissive than their older, white, or male counterparts (Ballew et al., 2023). In addition to demographic variables, human values and political orientation have been shown to be especially important predictors of climate change perception and worry (Gregersen et al., 2020).

The way people perceive and respond to climate change differs not only across different socio-cultural contexts within countries, but also across countries and regions. For example, segmentation studies similar to Leiserowitz et al.’s (2023) Six Americas have in other parts of the world identified differing numbers of segments with specific profiles (e.g., Four Europes: Kácha et al., 2022; Four Irelands: Leiserowitz et al., 2022; Five Germanys: Metag et al., 2017; Seven Britains: Wang et al., 2020). Irrespective of the issue of climate change, respondents in different countries or regions tend to exhibit different emotions, possibly because they appraise emotion-relevant events differently (Scherer, 1997) or differ in emotional conventions (Fernández-Dols et al., 2007) or collective responses (De Rivera et al., 2007).

This suggests that people from different countries may differ in their emotional response to climate change and this in turn may be linked to different climate-related appraisals and behaviors. Differences between countries can be traced to historic and socio-political roots which will have an impact on typical national attitudes toward climate change, energy use, or sustainability (Arnold et al., 2016; Steentjes et al., 2017; Davidson and Kecinski, 2022). Thus, we will relate our investigation of national differences to pertinent cultural theories, in particular to Hofstede’s cultural values (Hofstede, 2001), to Inglehart’s cultural dimensions (Inglehart and Welzel, 2023), and to Schwartz’ cultural value orientations (Schwartz, 2006).

### 1.3. This study

In this study, we focus on five discrete climate emotions – worry, fear, outrage, hope, and guilt – and explore two research questions.

Research question 1 asks how these discrete emotions are related to various appraisals of climate change and to a set of climate behaviors. This approach is based on our general appraisal-theoretical framework, which assumes that emotions are elicited by particular appraisal patterns, and that discrete emotions may trigger specific behavioral tendencies. We will explore the relationships between emotions and appraisals, and in a next step between emotions and behaviors.

Research question 2 addresses country differences concerning emotional experiences in regard to climate change but also concerning appraisal patterns and behavioral tendencies. We will compare samples from France, Germany, Norway, and the United Kingdom with respect to respondents’ emotional responses to climate change, their appraisals of climate change concerning various perceptions and evaluations, and their readiness to engage in various forms of climate action. We will also explore whether different sociodemographic groups within countries differ in their emotional responses to climate change.

We use a dataset from the European Perceptions of Climate Change project (EPCC: Steentjes et al., 2017) to answer the two research questions. The EPCC project conducted an international survey among representative samples from four European countries investigating a broad range of perceptions and judgments on the themes of climate and energy. The nationally representative samples comprise about 1,000 respondents from France, Germany, Norway and the United Kingdom, respectively.

Arnold et al. (2016) describe our selection of the four countries to be surveyed and their contrasted energy policy profiles at that time. France is the world’s largest nuclear power producer which means that it has relatively low greenhouse gas emissions *per capita*. Germany is the largest country and economy of the European Union and committed to its *Energiewende* policy, which includes phasing out nuclear power and decarbonizing their power sector over time. Norway is both a major exporter of fossil energy in the form of oil and gas, and a country with a fully renewable electricity sector consisting of hydropower, much of it dispatchable. The United Kingdom has, like Norway, access to oil and gas resources in the North Sea. The United Kingdom’s strategy to reduce their energy sector emissions has so far consisted of switching from its historically dominant coal to gas, investing in renewable energy including offshore wind, relying on nuclear power and exploring additional technological solutions (e.g., carbon capture and storage).

While the questionnaire was not designed specifically to investigate appraisal-theoretical assumptions about climate emotions, it does include questions on appraisals, emotions, and behaviors that allow us to answer our two research questions. Specifically, in addition to measures of the five emotions already discussed, the questionnaire included ten appraisal items, and seven behavioral options.

Our data analysis is exploratory and correlational. The first research question will be addressed using canonical correlation analysis techniques aimed at identifying regularities in appraisal-emotion and emotion-behavior linkages. The second research question, using correspondence analysis techniques, focuses on national differences, but also explores variation across sociodemographic subgroups (defined by age, gender, education, and political orientation) within the national samples. The EPCC survey data will be complemented with external publicly available country-level data on cultural dimensions. Specifically, we will use Hofstede’s cultural values (Hofstede, 2001), Inglehart’s cultural dimensions (Inglehart and Welzel, 2023), and Schwartz’ cultural value orientations (Schwartz, 2006). These will be used to enrich the interpretation of country differences with respect to their cultural embeddedness.

## 2. Material and sample

### 2.1. Survey procedure and sample

Representative national samples were drawn from four countries: France, Germany, Norway, and the United Kingdom with about 1,000 participants aged 15 or older per country (*n_France_* = 1,010; *n_Germany_* = 1,001; *n_Norway_* = 1,004; *n_UK_* = 1,033; total *N* = 4,048). Fieldwork took place in the period June 1–17, 2016. Data collection was commissioned to the international social research company Ipsos Mori. In France, Germany, and the United Kingdom, the questionnaire was administered as part of a weekly face-to-face omnibus survey that took place in respondents’ own homes using Computer-Assisted Personal Interviewing (CAPI). The average length of the interviews across the three countries ranged from 22 to 28 min. A stratified random sample was drawn for each country and quotas set for sociodemographic variables such as age, gender, and region. In Norway, face-to-face interviewing is uncommon due to the highly dispersed population. Interviews in Norway were therefore conducted by telephone using Computer Assisted Telephone Interviewing (CATI). Interviews in Norway lasted on average 24 min. Sampling source in Norway was the official telephone register, which includes all private non-anonymized numbers (30% of the registered numbers are landline and 70% mobile). A master version of the survey questionnaire was developed in English by the research team, which was subsequently double translated into French, German, and Norwegian by two independent teams of native speakers. Pilot interviews in all countries (total *N* = 231) allowed identification of potential issues with item formulations or translations before the field work was conducted *in extenso*. A detailed account of the survey methodology and sampling procedure is given in Steentjes et al. (2017).

### 2.2. Ethics approval

The study was approved by the Research Ethics Committee of the School of Psychology at Cardiff University. All respondents gave written informed consent in accordance with the Declaration of Helsinki. In addition, Ipsos Mori, the research company that administered the survey, adheres to the Market Research Society (MRS) code of conduct.

### 2.3. Measures

The questionnaire covered a broad range of topics including attitudes and judgments concerning climate change, climate policies, and energy options. The complete questionnaire is documented in Steentjes et al. (2017). In this study, we use the questions on (a) climate-related appraisals, (b) emotional reactions toward climate change, (c) climate-relevant behaviors, and (d) socio-demographic background variables. Most questions were accompanied by a five-point response scale and an additional “Do not know” option, which was treated as a missing value in the analyses. Questions that imply that the respondent acknowledges the existence of climate change were not posed to respondents who had previously chosen the option “there is no such thing as climate change;” (see Section 2.3.1). The measures are detailed in the following section, with a general overview provided in Table 1.

**Table 1:** Overview of measures.

Construct	Short label	Item and range	*N*	*M*	*SD*
**Climate change appraisals**					
Natural versus human causation	Causation	“Thinking about the causes of climate change, which, if any, of the following best describes your opinion?” 1 = Natural … 5 = Human	3,905	3.47	0.91
Perceived impact of climate change	Impact	“Overall, how positive or negative do you think the effects of climate change will be on <*France/Germany/Norway/United Kingdom*>?” 1 = Positive … 5 = Negative	3,850	3.76	0.91
Psychological distance (closeness): temporal distance (closeness)	tempDist	“When, if at all, do you think <*France/Germany/Norway/United Kingdom*>?” will start feeling the effects of climate change?”* 1 = already … 7 = never Recoded to: 1 = Distant future … 5 = Already now	3,926	4.18	1.24
Psychological distance (closeness): social distance (closeness)	socDist	“Climate change is likely to have a big impact on people like me.” 1 = Disagree … 5 = Agree	3,889	3.45	1.18
Psychological distance (closeness): geographical distance (closeness)	geoDist	“The impacts of climate change are mostly going to be felt in other countries.” ** 1 = Disagree … 5 = Agree Reverse coded to: 1 = Agree … 5 = Disagree	3,879	2.40	1.22
Injunctive norm	injNorm	“I feel that helping to tackle climate change is something that is NOT expected of me” ** 1 = disagree … 5 = agree Reverse coded to: 1 = Agree … 5 = Disagree	3,963	3.52	1.30
Descriptive norm	desNorm	“Most people around me take personal action to help tackle climate change.” 1 = Disagree … 5 = Agree	3,917	2.94	1.19
Perceived collective efficacy	Efficacy	“I am confident that, together, people in *<France/Germany/Norway/United Kingdom>* can make a difference when it comes to climate change.” 1 = Disagree … 5 = Agree	3,888	3.54	1.17
Moral concerns about climate change	moralConcern	“To what extent, if at all, do you have moral concerns about climate change?” 1 = Not at all … 5 = Very much	3,959	3.04	1.19
Technology optimism	technoSolve	“Science and technology will eventually solve our problems with climate change.” 1 = Disagree … 5 = Agree	3,834	2.99	1.17
**Climate emotions**					
Worry	Worry	“How worried, if at all, are you about climate change?” 1 = Not at all … 5 = Extremely	4,020	2.99	1.07
Hope	Hope	“When you think about climate change and everything that you associate with it, how strongly, if at all, do you feel each of the following emotions?” <*hope/fear/outrage/guilt*> 1 = Not at all … 5 = Very much	3,930	2.50	1.08
Fear	Fear	As above	4,002	2.47	1.19
Outrage	Outrage	As above	3,966	2.54	1.29
Guilt	Guilt	As above	3,976	2.15	1.09
**Climate change behaviors**					
Willingness to engage in individual private climate action: reduce energy use	reduceEnergy	“I am prepared to greatly reduce my energy use to help tackle climate change.” 1 = Disagree … 5 = Agree	3,989	3.66	1.17
Preparedness to individual social action: discuss with others	needDiscuss	“I do not feel the need to discuss my views on climate change with others”** 1 = Disagree … 5 = Agree Reverse coded to: 1 = Agree … 5 = Disagree	4,009	3.11	1.31
Preparedness to individual social action: challenge others	wouldChallenge	“I would challenge someone who says they do not care about climate change.” 1 = Disagree … 5 = Agree	3,989	3.23	1.34
Support for national climate policies: Increasing taxes on fossil fuels	polTaxes	“Various policies might be used to reduce climate change or deal with its effects. To what extent do you support or oppose the following policies in <*France/Germany/Norway/United Kingdom*>?” <*policy*> 1 = Oppose … 5 = Support	3,983	2.80	1.35
Support for national climate policies: Subsidizing renewable energy	polRenew	As above	3,999	4.00	1.10
Support for national climate policies: Increasing price of electricity	polPrice	As above	3,999	2.24	1.21
Support for national climate policies: Subsidizing insulation of homes	polInsulation	As above	3,981	3.70	1.14
Support for national climate policies: Banning non-energy-efficient appliances	polBan	As above	3,972	3.49	1.24
Support for national climate policies: Spending public money to prepare country for climate change	polPrepare	As above	3,989	3.94	1.06
Support for national climate policies: Giving public money to developing countries	polDevcountry	As above	3,991	3.53	1.21
Support for international climate agreements: Paris agreement	supportParis	“Do you support or oppose <*France/Germany/Norway/United Kingdom*>?” being part of the Paris Agreement?” 1 = Oppose … 5 = Support	3,910	4.09	1.03
Support for international climate agreements: economic penalties for violators	punishViolators	“Do you support or oppose introducing high economic penalties for countries that refuse being part of this agreement?” 1 = Oppose … 5 = Support	3,873	3.67	1.17
**Socio-demographic background variables**					
Age	Young Old	Ten-year intervals, 1 (15–24) … 5 (55–64), 6 (65+). Dichotomized to: Young = 15–44 years Old = 45+ years			
Gender	Male Female	1 = Male 2 = Female			
Education	Yes No	Yes = University degree No = No university degree			
Political orientation	Left Right	0 = left … 10 = right Dichotomized to: Left = 0–5 Right = 6–10			

Short Label is used in most text and figures. N indicates effective sample size without missing values. M: Arithmetic mean. SD, standard deviation. Some items were recoded, for a full description of items and response scales see Section 2.3. Means in the table refer to recoded scales.

*Recoded to 5-point scale by combining response categories 4, 5, and 6, and reverse coded.

**Reverse coded.

#### 2.3.1. Climate change appraisals

##### 2.3.1.1. Natural versus human causation

Respondents indicated whether they perceived climate change to be of natural or human origin by responding to the following question: “Thinking about the causes of climate change, which, if any, of the following best describes your opinion?” The following response options were presented: 1 (*climate change is entirely caused by natural processes*), 2 (*climate change is mainly caused by natural processes*), 3 (*climate change is partly caused by natural processes and partly caused by human activity*), 4 (*climate change is mainly caused by human activity*), 5 (*climate change is completely caused by human activity*). Responses to the additional response option “*There is no such thing as climate change*” were treated as a missing value in the analyses.

##### 2.3.1.2. Perceived impact of climate change

Perceived impact of climate change was measured with the following question: “Overall, how positive or negative do you think the effects of climate change will be on *<France/Germany /Norway/United Kingdom>*? The response scale ranged from 1 (*entirely positive*) to 5 (*entirely negative*). This question was not posed to respondents who had indicated in the perceived causation question that they believed that “there is no such thing as climate change.”

##### 2.3.1.3. Psychological distance (closeness)

Three dimensions of psychological distance were measured: temporal, social, and geographic distance. For the analysis, the responses were coded so that higher numbers correspond to greater perceived *closeness* of climate change (see Table 1).

*Temporal distance* (reverse coded) was tapped into by asking all respondents “When, if at all, do you think *<France/Germany /Norway/United Kingdom>* will start feeling the effects of climate change?.” Respondents were to choose from the following response options: 1 (*we are already feeling the effects*), 2 (*in the next 10 years*), 3 (*in the next 25 years*), 4 (*in the next 50 years*), 5 (*in the next 100 years*), 6 (*beyond the next 100 years*), 7 (*never*).

Two agree-disagree scales targeted social and geographical distance. *Social distance* was measured with the statement “Climate change is likely to have a big impact on people like me,” *geographical distance* (reverse coded) with the statement “The impacts of climate change are mostly going to be felt in other countries.” The response scale ranged from 1 (*strongly disagree*) to 5 (*strongly agree*). The questions on social and geographical distance were not posed to respondents who had indicated in the perceived causation question that they believed that “there is no such thing as climate change.”

##### 2.3.1.4. Norms

Two types of perceived norms were measured with agree-disagree scales. The injunctive norm question asked for the respondent’s level of agreement with the statement (reverse coded) “I feel that helping to tackle climate change is something that is NOT expected of me.” The statement for perceived descriptive norm read “Most people around me take personal action to help tackle climate change.” The response scale ranged from 1 (*strongly disagree*) to 5 (*strongly agree*) and was coded so that higher numbers reflect stronger perceived norms.

##### 2.3.1.5. Perceived collective efficacy

An agree-disagree scale asked for respondents’ perceived collective efficacy with the statement “I am confident that, together, people in *<France/Germany /Norway/United Kingdom>* can make a difference when it comes to climate change.” The response scale ranged from 1 (*strongly disagree*) to 5 (*strongly agree*). This question was not posed to respondents who had indicated in the perceived causation question that they believed that “there is no such thing as climate change.”

##### 2.3.1.6. Moral concerns about climate change

Respondents indicated the extent to which they had moral concerns about climate change. The question was introduced with the following passage: “Some people have moral concerns about climate change. For example, because they think that its harmful impacts are more likely to affect poorer countries, or because they feel a moral responsibility towards future generations. To what extent, if at all, do you have moral concerns about climate change?” Responses were given on a scale from 1 (*not at all*) to 5 (*very much*).

##### 2.3.1.7. Technology optimism

An agree-disagree scale asked respondents to indicate how much they agreed that “Science and technology will eventually solve our problems with climate change.” The response scale ranged from 1 (*strongly disagree*) to 5 (*strongly agree*).

#### 2.3.2. Climate emotions

Respondents were asked to rate the intensity with which they experienced the following five discrete emotions with respect to climate change: worry, hope, fear, outrage, and guilt.

Worry was assessed with the question: “How worried, if at all, are you about climate change?.” Responses were given on a five-point scale with the following options: 1 (*not at all worried*), 2 (*not very worried*), 3 (*fairly worried*), 4 (*very worried*), 5 (*extremely worried*).

The other four emotions were assessed in a single question, phrased “When you think about climate change and everything that you associate with it, how strongly, if at all, do you feel each of the following emotions?.” The four emotions hope, fear, outrage, and guilt were then listed, each accompanied by a five-point rating scale with the scale points 1 (*not at all*), 2 (*a little*), 3 (*moderately*), 4 (*quite a bit*), 5 (*very much*).

#### 2.3.3. Climate change behaviors

We considered four different types of climate-relevant actions which are akin to, but somewhat different from, the types of environmentally significant behaviors distinguished by Stern (2000).

Willingness to engage in *individual private climate action* was measured with an agree-disagree scale asking to which degree the respondents agreed with the statement “I am prepared to greatly reduce my energy use to help tackle climate change.” This question was not presented to respondents who had indicated that “there is no such thing as climate change” in the perceived causation question. The response scale ranged from 1 (*strongly disagree*) to 5 (*strongly agree*).

Preparedness to *individual social climate action* was measured with two agree-disagree statements: (a) “I do not feel the need to discuss my views on climate change with others” (reverse coded), and (b) “I would challenge someone who says they do not care about climate change.” Responses to both statements were given on a scale from 1 (*strongly disagree*) to 5 (*strongly agree*).

Support for *national climate policies* was elicited with the question “To what extent do you support or oppose the following policies in *<France/Germany /Norway/United Kingdom>*?,” followed with a list of eight policies: (a) increasing taxes on any use of fossil fuels (such as coal, oil, diesel, petrol, gas), (b) including nuclear power in the energy mix, (c) using public money to subsidise renewable energy such as wind and solar power, (d) increasing the price of electricity to reduce our consumption, (e) using public money to subsidise insulation of homes, (f) a law banning the sale of household appliances that are not energy efficient, (g) spending public money now to prepare the country for the impacts of climate change (e.g., building flood defenses), (h) giving public money to developing countries to help them deal with extreme weather, such as flooding and drought. Each policy was presented with a five-point response scale ranging from 1 (*strongly oppose*) to 5 (*strongly support*). The item on nuclear power (Item b) was later omitted from all analyses because it was found to be an outlier; it is therefore not listed in Table 1.

Support for *international climate agreements* was tapped into with two questions concerning the Paris Agreement: (a) “In Paris in December 2015, most countries agreed to an international agreement that aims to keep global temperature rises below 2 degrees. Do you support or oppose *<France/Germany /Norway/United Kingdom>* being part of this agreement?” and (b) “Do you support or oppose introducing high economic penalties for countries that refuse being part of this agreement?” Both questions were presented with a five-point response scale ranging from 1 (*strongly oppose*) to 5 (*strongly support*).

#### 2.3.4. Socio-demographic background variables

We included four socio-demographic background variables: age, gender, education, and political orientation. These variables have been shown in the literature to predict climate change appraisals. For example, a meta-analysis of 171 studies across 56 countries by Hornsey et al. (2016) demonstrated that people who believe in the existence of climate change tend to be younger, female, more highly educated, and oriented toward the left side of the political spectrum.

Age was measured in six 10-year intervals: 1 (*15–24*), 2 (*25–34*), 3 (*35–44*), 4 (*45–54*), 5 (*55–64*), 6 (*65+*). Gender was categorized as *male* (1) or *female* (2). Education distinguished respondents who held a completed university degree (1) from those who did not (0).

The following question was used to tap into political orientation: “In politics people sometimes talk of ‘left’ and ‘right’. Using a scale from 0 to 10, where 0 means the left and 10 means the right, where would you place yourself on this scale?”

### 2.4. Analytical approach

With respect to our fist research question, we use canonical correlation analysis (Thompson, 1984, 1991; Sherry and Henson, 2005; Tabachnick and Fidell, 2013) to analyze the relations between appraisals and emotions on the one hand, and between emotions and climate behaviors on the other hand. In canonical correlation analysis, two sets of variables, for example, a set X and a set Y, are correlated: for each set X and Y, a linear function of the respective variables is constructed with weights that maximize the correlation between the resulting linear functions of the X and the Y variables. The linear functions are called canonical variates, and the correlation between a pair of canonical variates is called the canonical correlation. If the canonical correlation is substantive, the weights can be interpreted as in regression analysis. That is, large weights indicate that the respective variables contribute strongly to the canonical variate, and thus to the correlation with the variables of the other set. It is common practice to refer also to the structural coefficients, that is, the (squared) correlation between a variable and its canonical variate, for interpretation (Sherry and Henson, 2005). Usually, more than one canonical correlation can be computed between two sets X and Y, with the second (and further) canonical correlations being based only on the variance not accounted for by the first (or preceding) canonical correlations (for details see Thompson, 1984, 1991). In our analysis, the sets X and Y are represented by the set of emotions and the set of appraisals, and by the set of emotions and the set of behaviors, respectively. The first assumption is that there is a systematic relationship between specific appraisals and specific emotions, and the second assumption is that there is a relationship between specific emotions and specific behaviors. We examine these two assumptions in a purely correlational manner, since due to the study design causal inferences are impossible. Still, correlational analyses can yield cues about the strength and directions of assumed causal relations.

Our second research question is addressed by analyzing country differences with respect to emotions, appraisals, and behaviors, primarily based on analyses of variance and correspondence analyses. We analyze country differences in three steps. In Step 1, we explore whether participants in different countries show distinctive patterns of climate emotions, of appraisals of climate change, and of behavioral responses to climate change. The next two steps serve to explore country differences in more depth. These steps focus on emotions; the respective analyses for appraisals and behaviors can be found in Supplementary material. In Step 2, we look at within-country differences concerning emotional reactions to climate change. Specifically, we explore to what extent subgroups that are defined by sociodemographic and other background variables within each country differ concerning their patterns of emotional reactions. In Step 3, we analyze how the country-specific patterns of emotional response (Step 1) are related to country-level cultural dimensions. For Step 3, we used publicly available country-level data on cultural dimensions, such as Hofstede’s (2001) cultural values, plus political orientation from our own data set and fitted these dimensions as supplementary variables into the configuration of emotional responses obtained in Step 1.

Across these three steps, we conducted a series of correspondence analyses (Nenadic and Greenacre, 2007; Greenacre, 2016). Correspondence analysis is commonly used to analyze contingency tables of categorical variables; in particular, it yields a geometric visualization of the relationships between the relative frequency profiles of categories (for details, see Greenacre, 2016).

In our first correspondence analysis, we investigated relationships between emotions and countries. Here, we consider country as well as emotion as categorical variables with four and five categories, respectively; we compute the mean of each emotion for each country (Table 2A), obtaining emotion profiles for each country. Correspondence analysis geometrically depicts the similarity between the emotion profiles of the four countries, as well as the profiles of each emotion across the four countries in a low-dimensional space (commonly in two dimensions). As we have arithmetic means instead of frequencies, we convert the means into pseudo-contingencies suitable for correspondence analysis; specifically, the frequency of a Nation A co-occurring with an Emotion E is computed as *f_A,E_ = mean_A,E_ × 100*, truncated to an integer value without decimals (e.g., the mean worry of 2.69019 in the United Kingdom will yield a pseudo-contingency of 269). The two-dimensional configurations then inform us about dissimilarities between countries with respect to their emotional profiles.

**Table 2:** Means of emotion ratings (A), appraisal ratings (B), and behavior ratings (C) by country.

Variable	France	Germany	Norway	United Kingdom
**(A) Emotion ratings**				
Worry	3.28	2.99	3.01	2.69
Hope	2.30	2.62	2.59	2.49
Fear	2.71	2.78	2.11	2.30
Outrage	3.04	2.90	1.93	2.28
Guilt	2.17	2.38	2.04	2.03
**(B) Appraisal ratings**				
Causation	3.61	3.59	3.29	3.39
Impact	3.91	4.02	3.55	3.56
tempDist	4.27	4.14	4.17	4.16
socDist	3.67	3.30	3.63	3.18
geoDist	1.96	2.77	2.28	2.63
injNorm	3.76	3.12	3.62	3.58
desNorm	3.10	2.74	3.13	2.80
Efficacy	3.77	3.29	3.57	3.50
moralConcern	3.24	2.79	3.03	3.07
technoSolve	2.69	2.84	3.32	3.11
**(C) Behavior ratings**				
needDiscuss	3.34	2.90	3.20	3.00
wouldChallenge	3.47	2.81	3.38	3.25
reduceEnergy	4.03	3.42	3.69	3.51
supportParis	4.11	3.99	4.32	3.95
punishViolators	3.97	3.73	3.40	3.58
policy_1publSubs	3.70	3.49	3.78	3.62
policy_2indiCost	2.06	2.13	2.66	2.46

See Table 1 for the meaning of variable labels. Behavior index variables: policy_1publSubs is the index variable representing policies focusing on subsidies; policy_2indiCost is the index variable representing policies that implicate an increase in individual costs.

In an analogous way we proceed with the analysis of appraisal and climate behavior differences between countries and with the analysis of within-country subgroups.

Canonical correlation analysis and correspondence analysis are both principally descriptive and exploratory. Whenever we report null hypothesis tests and *p*-values, note that even small effects sizes are likely to be significant due to the large sample size (depending on the analysis and variables used, the effective sample size varies due to missing values, but usually comprises about 900 respondents per country).

## 3. Results

Research question 1 asks about the relationships between emotions, appraisals, and behavioral intentions. Theoretically, the three groups of variables (appraisals, emotions, behaviors) yield two pairs of variable sets: Appraisals paired with emotions, and emotions paired with behaviors. Using canonical correlation analysis, we search for relationships between distinct variables of one set, that is, appraisals or emotions, with distinct variables of the second set, that is, emotions or behaviors, respectively.

Research question 2 refers to differences between countries with respect to emotions, appraisals, and behaviors. Using correspondence analysis, we describe the relative differences and similarities between France, Germany, Norway, and the United Kingdom. We augment these findings by (i) examining within-country differences with respect to several sociodemographic variables, and (ii) by adding contextual cultural dimensions to the analyses.

All analyses were done with the R computing environment (R Core Team, 2023), version 4.2.3, using the package *ca* (Nenadic and Greenacre, 2007) for correspondence analysis and the package *CCA* (González and Déjean, 2021) for canonical correlation analysis.

Supplementary Table S1 in Supplementary material shows for all measures the country means and confidence intervals, along with the number of cases and standard deviations.

### 3.1. Relating emotions to appraisals and to behaviors

First, we examine the relationships between the set of appraisals and the set of emotions, and second, the relationship between emotions and the set of behaviors.

There is evidence in the literature that the relationship between appraisals and emotions is likely reciprocal with feedback loops (van der Linden, 2014). This means that emotions not only arise from the judgment and decision-making process, but may also form the basis for construing and appraising a decision in ways that are in line with the emotional state (Lerner et al., 2015). We do not want to exclude this possibility, and our correlational analysis allows an interpretation of results as indicating a bi-directional relationship. For the sake of simplicity, however, we will in the following discuss appraisals as the sources of emotions, whereas in the second analysis emotions are assumed to motivate behaviors.

#### 3.1.1. Associations between appraisals and emotions

Table 3 shows the bivariate Pearson correlations between the set of ten appraisal variables and the set of five emotion variables. The correlations suggest, to point out a few examples, that stronger belief in human causation of climate change may lead to worry, fear, outrage, and guilt; and that hope correlates negatively with perceived impact of climate change but positively with, for example, perceiving high collective efficacy and believing in technological solutions.

**Table 3:** Correlations between appraisals and emotions.

Appraisal	Worry	Hope	Fear	Outrage	Guilt
Causation	0.35	−0.02	0.27	0.29	0.23
Impact	0.30	−0.12	0.25	0.27	0.16
tempDist	0.31	−0.00	0.21	0.18	0.14
socDist	0.40	0.04	0.35	0.28	0.27
geoDist	−0.07	0.02	−0.03	−0.03	−0.01
injNorm	0.24	−0.01	0.14	0.13	0.15
desNorm	0.15	0.14	0.09	0.09	0.09
Efficacy	0.22	0.17	0.12	0.11	0.13
moralConcern	0.47	0.12	0.41	0.37	0.38
technoSolve	−0.06	0.14	−0.08	−0.12	0.04

For the distance variables socDist, geoDist, and tempDist larger values represent judgments of closeness (refer to Section 2 measures concerning the coding of variables). See Table 1 for the meaning of the appraisal labels.

A canonical correlation analysis yields five canonical correlations (analogous to principal component analysis, canonical correlations are necessarily successively smaller): 0.67, 0.31, 0.14, 0.12, 0.08; we consider only the first two correlations as substantial, accounting for 54.6% of the covariance among the two sets of variables (due to the large sample size, all canonical correlations are significant, with *p* < 0.001).

The first canonical variate for the appraisal set is mainly determined by causation, social distance (closeness), and moral concern; Table 4 shows the standardized weights and the squared structural coefficients (correlation between variable and canonical variate). These judgments all reflect human involvement and moral responsibility, and are somewhat related to so called deontological judgments (Böhm and Pfister, 2000; Böhm, 2003). The first canonical variate for the emotion set is mainly determined by worry, fear, and outrage, and somewhat less by guilt (Table 5), all representing negative emotions.

**Table 4:** Standardized function weights and (squared) structural coefficients of appraisal variables for the first and second canonical variate.

Appraisal	First canonical variate		Second canonical variate	
	w_A,CV1_	*r* ^2^_A,CV1_	w_A,CV2_	*r* ^2^_A,CV2_
Causation	**0.24**	**0.28**	−0.11	0.01
Impact	0.22	0.21	**−0.48**	**0.19**
tempDist	0.15	0.21	0.01	0.00
socDist	**0.31**	**0.43**	−0.03	0.01
geoDist	−0.04	0.01	0.17	0.01
injNorm	0.08	0.13	−0.12	0.00
desNorm	0.10	0.05	0.28	0.19
Efficacy	0.04	0.10	**0.42**	**0.33**
moralConcern	**0.53**	**0.66**	0.29	0.10
technoSolve	−0.10	0.01	**0.48**	**0.42**

The column w_A,CV1_ depicts the standardized function weights of each appraisal for the first canonical variate (CV1), and the column *r*^2^_A,CV1_ depicts the squared correlation between the respective appraisal and the first canonical variate (structural coefficient). The same applies to columns w_A,CV2_ and *r*^2^_A,CV2_ with respect to the second canonical variate (CV2). Appraisals with the three largest weights are in bold. Canonical variates are coded so that correlations with typical variables are positive. See Table 1 for the meaning of the appraisal labels.

**Table 5:** Standardized function weights and (squared) structural coefficients of emotion variables for the first and second canonical variate.

Emotion	First canonical variate		Second canonical variate	
	w_E,CV1_	*r* ^2^_E,CV1_	w_E,CV2_	*r* ^2^_E,CV2_
Worry	**0.63**	**0.81**	0.13	0.00
Hope	−0.02	0.01	**0.89**	**0.78**
Fear	**0.24**	**0.59**	−0.14	0.02
Outrage	**0.20**	**0.52**	**−0.41**	**0.09**
Guilt	0.17	0.39	**0.35**	**0.05**

The column w_E,CV1_ depicts the standardized function weights of each emotion for the first canonical variate (CV1), and the column *r*^2^_E,CV1_ depicts the squared correlation between the respective emotion and the first canonical variate (structural coefficient). The same applies to columns w_E,CV2_ and *r*^2^_E,CV2_ with respect to the second canonical variate (CV2). Emotions with the three largest weights are in bold. Canonical variates are coded so that correlations with typical variables are positive.

The first canonical correlation of 0.67 between appraisals and emotions suggests that, presuming a directional influence, appraisals of human involvement and responsibility primarily elicit negative emotions in the context of climate change, in particular worry, fear, and outrage. Note that impact also has a weight of 0.22, suggesting that appraisals of human involvement go together with an expectation of strong negative impact of climate change.

The second canonical correlation of 0.31 between appraisals and emotions is clearly smaller than the first canonical correlation, but still substantial. The second canonical variate for appraisals implies large weights for efficacy and belief in technological solutions; also, the weight for impact is large and negative, suggesting that this variate is associated with a judgment that the impact of climate change will be less negative. These appraisals involve confidence in human agency and optimism with respect to climate change. Accordingly, the second canonical variate for emotions has a large weight for hope, and a negative weight for outrage, suggesting that hopeful feelings counteract feelings of outrage, possibly leading to a state of calmness and composure. Interestingly, we also find a moderately positive weight for guilt, suggesting that the belief that the climate crisis can be solved is associated with feelings of guilt; maybe this is due to thinking that not enough has been done or has been accomplished so far, though feasible in principle.

In sum, the canonical analysis of appraisals and emotions suggests that two bundles of appraisals are relevant in the context of climate change: judgments of human involvement and responsibility, and judgments of efficacy and technical feasibility. The first appraisal bundle elicits feelings of worry, fear, and outrage, whereas the second bundle of appraisals elicits feelings of hope, and, to a lesser extent, of guilt.

**Figure 1:**
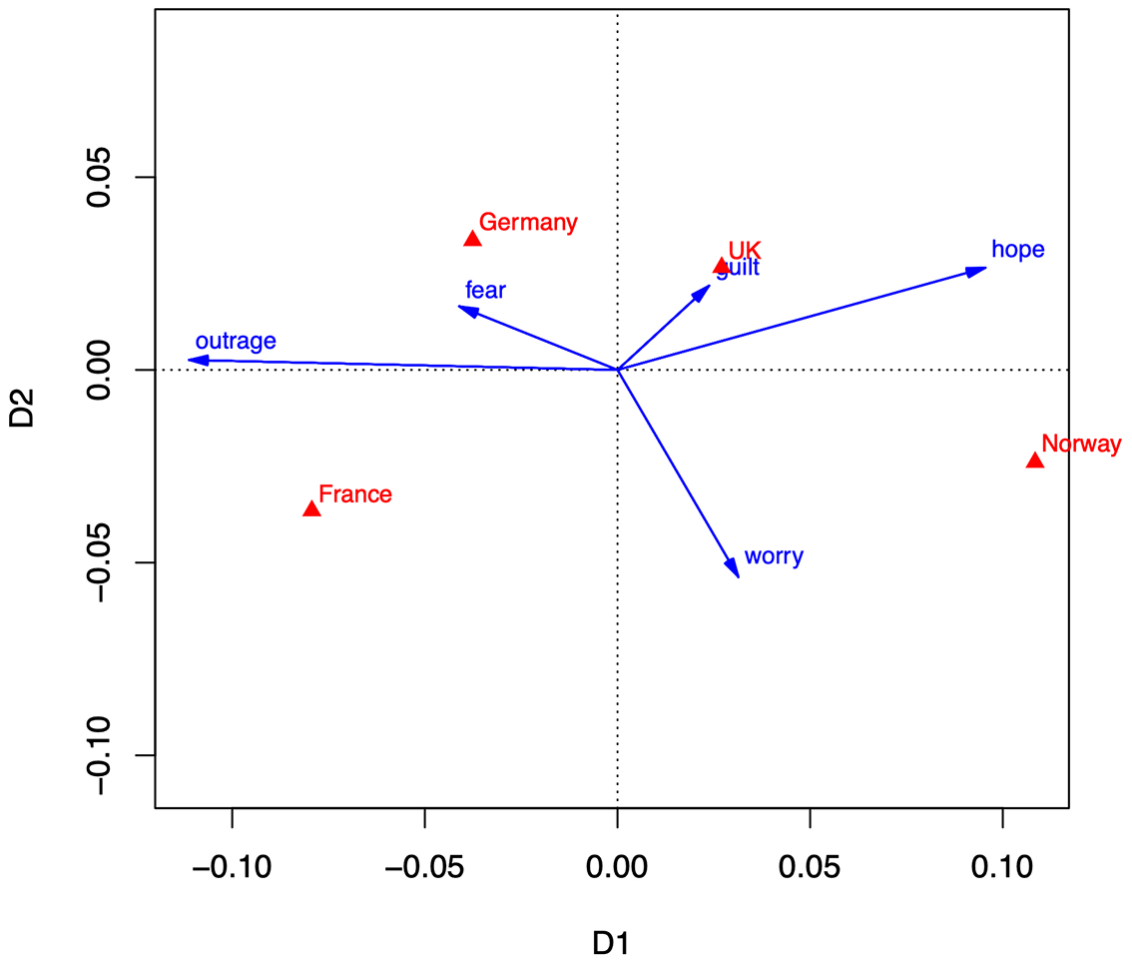
Correspondence analysis of emotion-country means. Dimension 1 accounts for 83% of variance. Dimension 2 accounts for 16% of variance. Emotions are depicted as blue vectors, countries as red triangles.

**Figure 2:**
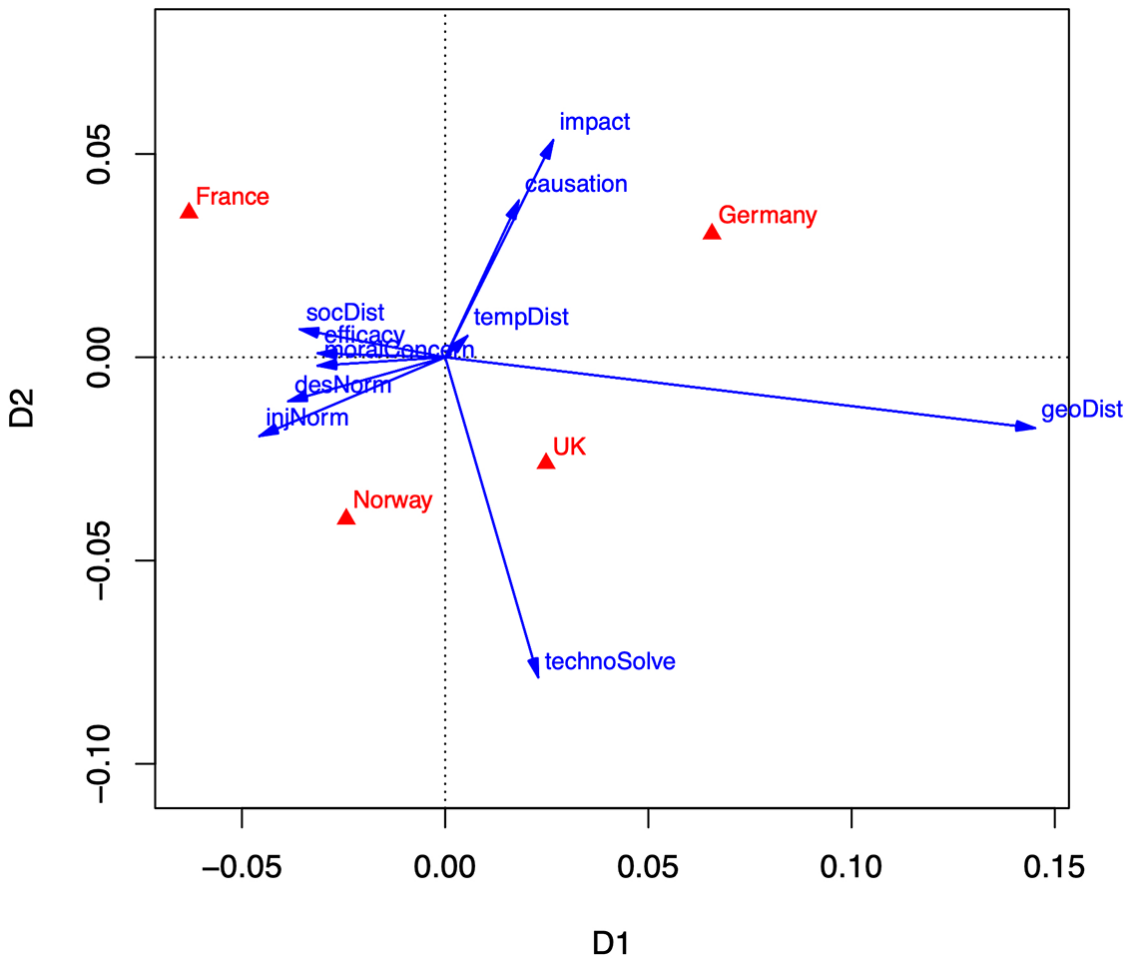
Correspondence analysis of appraisal-country means. Dimension 1 accounts for 63% of variance. Dimension 2 accounts for 29% of variance. Appraisals are depicted as blue vectors, countries as red triangles.

These findings largely parallel the findings from the correspondence analysis that will be reported in Section 3.2, where the correspondence between countries and emotions yields a contrast of fear and outrage with hope and guilt (see Figure 1), and a contrast of belief in human causation with a belief in technological solutions (see Figure 2).

Additionally, we compare the four countries on the canonical variates. Table 6A shows the means on the respective canonical covariates for the four countries. Along the lines of the interpretation provided above, we label the covariates of the first canonical correlation *Involvement* (appraisals) and *Worry/Outrage* (emotions), and those of the second canonical correlation *Agency* (appraisals) and *Hope/Calmness* (emotions). France turns out to be highest on Involvement, whereas the other countries are slightly below average; accordingly, France is also highest on Worry/Outrage, followed by Germany being also above average. With respect to Agency, Norway scores highest, followed by the United Kingdom; Norway also shows most intense feelings of Hope/Calmness, in contrast to France being clearly below average. Again, we will see parallel findings in Section 3.2.

**Table 6:** Means of countries on the first and second canonical variates of appraisals-emotions relation (A), and emotions-behaviors relation (B).

Canonical Variate	France	Germany	Norway	United Kingdom	
**(A) Appraisals and emotions**					
1 Appraisals	0.30	−0.10	−0.05	−0.14	Involvement
1 Emotions	0.30	0.15	−0.17	−0.27	Worry/Outrage
2 Appraisals	−0.13	−0.30	0.28	0.14	Agency
2 Emotions	−0.30	0.02	0.27	0.01	Hope/Calmness
**(B) Emotions and behaviors**					
1 Emotions	0.26	0.12	−0.10	−0.27	Worry/Outrage
1 Behaviors	0.25	−0.27	0.10	−0.08	Reduce/Discuss
2 Emotions	−0.38	−0.12	0.44	0.06	Hope/Calmness
2 Behaviors	−0.44	−0.12	0.43	0.13	Cost Increase

Canonical covariates are standardized variables with a mean of zero and a variance of one. Last column contains an interpretational label.

#### 3.1.2. Associations between emotions and climate behaviors

Table 7 shows the bivariate Pearson correlations between the set of five emotion variables and the set of seven behavior variables.

**Table 7:** Correlations between emotions and behaviors.

Behavior	Worry	Hope	Fear	Outrage	Guilt
needDiscuss	0.31	0.01	0.24	0.25	0.20
wouldChallenge	0.39	0.07	0.27	0.26	0.25
reduceEnergy	0.40	0.10	0.30	0.28	0.27
supportParis	0.31	0.06	0.17	0.13	0.16
punishViolators	0.30	0.02	0.24	0.25	0.19
policy_1publSubs	0.26	0.05	0.15	0.13	0.14
policy_2indiCost	0.27	0.08	0.18	0.14	0.29

See Table 1 for the meaning of the labels for the behaviors. policy_1publSubs: index variable representing policies focusing on subsidies. policy_2indiCost: index variable representing policies that implicate an increase in individual costs.

For the behaviors, we added two index variables, derived from the set of items investigating the degree of support for specific national climate policies. From the set of national policies (see Section 2.3.3.), we omitted the item asking about support for nuclear power, because this item shows no association with any of the other behaviors (consistent with previous studies, e.g., Böhm et al., 2018; Doran et al., 2019)‚ and can be considered an outlier; to avoid an undue distortion of the results, we excluded this item from analysis. For an analysis that focuses specifically on this variable, see Sonnberger et al., 2021.

A factor analysis of the remaining seven national policies yields two factors (promax rotation, 36.2% variance accounted for, factor correlation = −0.48). The first factor represents support for public subsidies such as grants for home insulation, the second factor represents support for policies increasing the individual costs for behaviors harmful for the climate (such as taxes on the use of fossil fuels). We created two index variables representing the factors by averaging the two best marker variables for each factor; ‘policy_1publSubs’ represents the first factor (support for public subsidies, computed as the mean of polRenew and polInsulation; see Table 1), and ‘policy_2indiCost’ represents the second factor (support for individual costs, computed as the mean of polTaxes and polPrice).

Note that all correlations are positive (Table 7), given that all behavior scales are coded so that higher values represent behavior being more in accordance with climate change engagement. For all behaviors (except individual cost policies), worry shows the highest correlation, followed by fear and outrage. Guilt shows correlations similar to fear and outrage, except for punishing violators of the Paris Agreement, where the correlation is lower, and for individual cost policies, where it is higher. Hope shows low correlations with all behaviors, the largest being 0.10 with the intention to reduce energy consumption.

A canonical correlation analysis (Table 8) yields five canonical correlations of 0.58, 0.19, 0.14, 0.05, and 0.02, with only the first three correlations being significant (*p* < 0.01); the first two correlations account for 77% of covariance, and hence we ignore the last three canonical correlations as insubstantial.

**Table 8:** Standardized function weights and (squared) structural coefficients of emotion variables for the first and second canonical variate.

Emotion	First canonical variate		Second canonical variate	
	w_E,CV1_	*r* ^2^_E,CV1_	w_E,CV2_	*r* ^2^_E,CV2_
Worry	**0.73**	**0.87**	0.22	0.01
Hope	0.07	0.03	**0.26**	**0.07**
Fear	**0.10**	**0.46**	−0.28	0.11
Outrage	**0.14**	**0.43**	**−0.91**	**0.47**
Guilt	0.23	0.41	**0.83**	**0.08**

The column w_E,CV1_ depicts the standardized function weights of each emotion for the first canonical variate (CV1), and the column *r*^2^_E,CV1_ depicts the squared correlation between the respective emotion and the first canonical variate (structural coefficient). The same applies to columns w_E,CV2_ and *r*^2^_E,CV2_ with respect to the second canonical variate (CV2). Emotions with the three largest weights are in bold. Canonical variates are coded so that correlations with typical variables are positive.

The first emotional canonical variate (Table 8) is mainly determined by worry, and to a lesser degree by fear, outrage, and guilt, as indicated by the squared structural coefficients. This bundle of negative emotions implies feelings of two aspects of negativity: Threat (worry and fear) as well as morality (outrage and guilt). The first canonical variate for behaviors (Table 9) is mainly determined by the intention to reduce energy consumption, and by the tendency to discuss climate change as well as to challenge other people who do not care about the issue. Also, the tendency to punish violators of the Paris Agreement contributes somewhat to the bundle constituting the first canonical variate of behaviors. Assuming that emotions motivate behaviors, the pattern of canonical variates suggests that strong feelings of worry, fear, outrage and guilt will trigger intentions to reduce one’s energy consumption, as well as elicit a motivation to discuss climate change and to challenge other people who do not care about climate change.

**Table 9:** Standardized function weights and (squared) structural coefficients of behavior variables for the first and second canonical variate.

Behavior	First canonical variate		Second canonical variate	
	w_B,CV1_	*r* ^2^_B,CV1_	w_B,CV2_	*r* ^2^_B,CV2_
needDiscuss	**0.26**	**0.36**	**−0.43**	**0.14**
wouldChallenge	**0.32**	**0.55**	−0.02	0.00
reduceEnergy	**0.39**	**0.55**	−0.09	0.00
supportParis	0.11	0.26	0.34	0.03
punishViolators	0.24	0.29	**−0.58**	**0.15**
policy_1publSubs	0.10	0.18	0.05	0.01
policy_2indiCost	0.18	0.27	**0.83**	**0.43**

The column w_B,CV1_ depicts the standardized function weights of each behavior for the first canonical variate (CV1), and the column *r*^2^_B,CV1_ depicts the squared correlation between the respective behavior and the first canonical variate (structural coefficient). The same applies to columns w_B,CV2_ and *r*^2^_B,CV2_ with respect to the second canonical variate (CV2). Behaviors with the three largest weights are in bold. Canonical variates are coded so that correlations with typical variables are positive. See Table 1 for the meaning of the labels for the behaviors. policy_1publSubs: index variable representing policies focusing on subsidies. policy_2indiCost: index variable representing policies that implicate an increase in individual costs.

The second emotional canonical variate (Table 8) implies large weights for feelings of guilt and hope, and for *not* feeling outrage, and also for *not* feeling fear. However, looking at the squared structural coefficients, the pattern is somewhat complex, because only outrage shows a substantial squared correlation with the second covariate (0.47), whereas hope, fear and guilt show fairly small squared correlations (0.07, 0.11, 0.08, respectively). Inhibiting outrage and fear, complemented with some feelings of guilt and hope, suggests a bundle of emotions expressing calmness and hopefulness. The second behavioral canonical variate (Table 9) is largely determined by the index variable for supporting strategies which imply price and tax increases. This support for increasing individual costs is associated with tendencies *not* to discuss with other people and *not* to punish violators of the Paris Agreement. Thus, the second behavioral function represents a focus on price effects and a tendency not to engage in social behaviors such as discussing with other people. Assuming that emotions are the relevant triggers of behavioral tendencies, we may maintain that feelings of hope and guilt as well as a general calmness without fear or outrage in the face of climate change will trigger behaviors that support policies targeted at increasing cost, but will reduce tendencies to discuss climate change issues or to punish violators of international agreements.

As we have done with the appraisal-emotion analysis, we compare the four countries on the canonical variates of the emotion-behaviors relationship. Table 6B shows the means obtained on the respective canonical covariates for the four countries. We label the two covariates of the first canonical correlation *Worry/Outrage* (emotions) and *Reduce/Discuss* (behaviors), and those of the second canonical correlation *Hope/Calmness* (emotions) and *Cost Increase* (behaviors). France and Germany show scores above average on the Worry/Outrage covariate, though only France also shows a substantial score on the Reduce/Discuss behavioral covariate; interestingly, Germany obtains the lowest score on that dimension. Norway and more so the United Kingdom score below average on the Worry/Outrage covariate. Concerning the second covariates, Norway displays by far the highest score on the Hope/Calmness covariate, followed by the United Kingdom with a neutral position. French and German respondents are not calm at all, which is in accord with the outcomes on the first covariate. Norwegians strongly support a policy of price and tax increases to tackle climate change, whereas French respondents strongly oppose such policies. These findings largely confirm the results from the correspondence analyses that will be reported in Section 3.2.

### 3.2. Differences between countries

#### 3.2.1. National patterns of emotional reactions to climate change

Do citizens in different countries respond emotionally in different ways to climate change? Table 2A shows the mean emotion ratings in France, Germany, Norway, and the United Kingdom for the five measured emotions. Mean ratings are mostly near the scale midpoint of three. According to a multivariate repeated measurement analysis of variance with emotion rating as dependent variable and Country and Emotion as independent variables, the effect of Country, the effect of Emotion, and as well the interaction effect are significant (*p* < 0.001 for all effects). The interaction effect in particular is an indication that each nation shows a distinctive emotion profile. However, effect sizes are rather small (Country 𝜂^2^ = 0.06, Emotion 𝜂^2^ = 0.10, Country × Emotion 𝜂^2^ = 0.05). Descriptively, worry is the most intense emotion in each country. For the United Kingdom and Norway, hope is second, whereas for France and Germany outrage is second. Note that, while all emotions were measured on five-point rating scales, the labels of the worry scale differ from those of the other emotions, so that the means are not precisely comparable (the label for the scale endpoint was more extreme for worry with 5 = *extremely*, than the label for the other emotions with 5 = *very much*); that worry still has the highest mean of all emotions in all countries can therefore be taken as an indication that worry is indeed the most endorsed emotion.

To obtain a more nuanced picture, Figure 1 shows the two-dimensional representation of country and emotion profiles according to a correspondence analysis of the emotion profiles (see Section 2.4). Emotions are represented as vectors, countries as points (depicted as triangles). For interpretation, country points can be projected onto the emotion vectors, indicating, for example, that France is high on outrage, whereas Norway is high on hope. Note that the geometric relationships are based on relative frequencies; for example, although all countries have largest means for worry, Germany has a relatively high mean for fear, compared to other countries. Thus, the specifics of each country profile relative to other countries are highlighted in correspondence analysis. The horizontal axis (Dimension 1, accounting for 83% of variance) might be interpreted as an outrage versus hope dimension, onto which the countries project in the order France, Germany, United Kingdom, and Norway from left (outrage) to right (hope). The vertical axis (Dimension 2, 16% of variance) is mainly characterized by worry, with Germany and the United Kingdom showing relatively lower and Norway and France relatively higher amounts of worry.

In sum, emotional responses to climate change in the context of the four countries can be largely represented on a dimension going from outrage to hope, accounting for a large part of the variance. Nations are clearly ordered on that dimension with France and Germany located at the outrage/fear end, and United Kingdom and Norway at the hope end. A second, though less important, dimension indicates amounts of worry, with Norway and France expressing more worry than the United Kingdom and Germany. National differences with respect to these dimensions are obvious in this display, but note that the absolute effect of countries in explaining emotional differences is small (𝜂^2^ = 0.05 according to the analysis of variance reported above).

#### 3.2.2. National patterns of appraisals of climate change

Table 2B shows the mean ratings on the ten appraisal scales for each country. While there is some variation, most means are relatively close to the scale mean of three on a five-point scale; an exception is the tempDist item (temporal distance/closeness) which has means above four, indicating that most people think that the effects of climate change are timewise very near or already happening. An analysis of variance with appraisal rating as dependent variable and Country and Appraisal as independent variables (with Appraisal as repeated measurement variable) yields significant main and interaction effects (*p* < 0.001 for all effects). Effect sizes are 𝜂^2^ = 0.10 for Country, 𝜂^2^ = 0.19 for Appraisal, and 𝜂^2^ = 0.04 for the Country × Appraisal interaction.

Following the same procedure as for the emotion ratings, we transformed the appraisal rating means to pseudo-contingencies, which served as input to a correspondence analysis. The two-dimensional configuration is depicted in Figure 2. The first dimension accounts for 63% of the variance, while the second dimension accounts for 29%.

The horizontal axis in Figure 2 shows a contrast between geographical distance on the right and social distance on the left. Note that distance variables (geoDist, socDist, tempDist) are coded so that high values indicate closeness and low values indicate remoteness. Hence, high values denote that climate change is felt also in the respondent’s country (geoDist), that climate change is soon or already happening (tempDist), and that climate change involves also people like ‘me’, the respondent (socDist). Projections of the countries on the horizontal axis suggest that German respondents focus their appraisal more on geographical closeness, and French respondents more on social closeness (with United Kingdom and Norwegian respondents in-between). Furthermore, social distance is strongly associated with ratings of efficacy, moral concern, injunctive and descriptive norms, indicated by small angles between the respective vectors, and all generally pointing into the same direction as socDist. That is, respondents who judge themselves as being affected by climate change (social closeness) also evaluate collective efficacy as relatively high and express more moral concern and more compliance with moral norms requesting to mitigate climate change. France and Norway show especially strong appraisals in that direction, Germany and the United Kingdom less so. The vertical axis distinguishes between appraisals that focus on the human causation of climate change and its negative impacts (pointing upwards), with German and French respondents endorsing these judgments, and appraisals focusing on technological solutions (pointing downwards), with Norwegian and United Kingdom respondents endorsing this aspect.

In sum, in the context of the four nations investigated, we observe a bundle of strongly associated appraisal aspects all related to personal involvement (social closeness, moral considerations, efficacy), in contrast to an appraisal focus on the geographical distance of climate change effects. Temporal distance plays no differentiating role, which may be due to the fact that a majority of 62% of the respondents think that we are already feeling the effects of climate change, and only a minority of 6% think that effects will be felt only in the very distant future, if at all. Additionally, people may focus either on human causation and negative impacts of climate change, or on the technological and scientific solutions. Clear national differences in appraisal patterns emerge between France, Germany, the United Kingdom, and Norway on both dimensions, though the overall effect of countries is small (𝜂^2^ = 0.04).

#### 3.2.3. National patterns of climate behavior

Table 2C shows the mean ratings for seven behavioral options. The behavior variables include two index variables concerning policy support for public subsidies and individual price increase (see Section 3.1.2).

Most means are near or above the scale midpoint of three, except support for policies that would increase individual costs. An analysis of variance with behavioral rating as dependent variable and Behavior and Country as independent variables (analogous to the previous analyses) again yields significant main and interaction effects (all *p* < 0.001). Effect sizes are 𝜂^2^ = 0.04 for Country, 𝜂^2^ = 0.22 for Behavior, and 𝜂^2^ = 0.04 for the Country × Behavior interaction.

**Figure 3:**
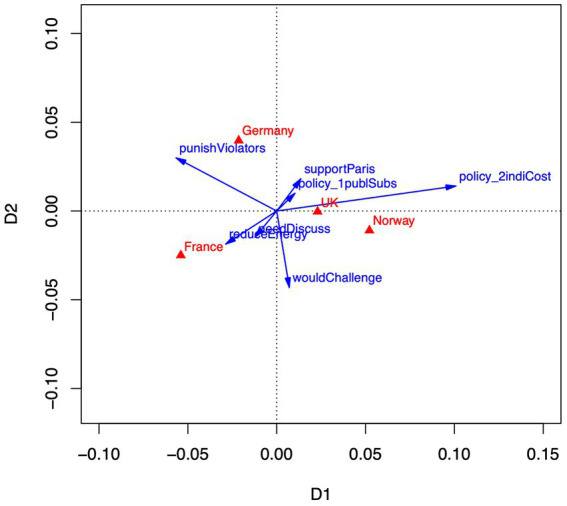
Correspondence analysis of behavior-country means. Dimension 1 accounts for 71% of variance. Dimension 2 accounts for 29% of variance. Behaviors are depicted as blue vectors, countries as red triangles.

Figure 3 depicts the two-dimensional configuration from a correspondence analysis with seven behavioral options. The first dimension accounts for 71% of variance, the second dimension accounts for 24%. Roughly, the horizontal axis shows a contrast between support for punishing nations that violate the Paris Agreement, and support for increasing individual costs for harmful energy use. Whereas German and French respondents tend to favor political punishment, Norwegian and United Kingdom respondents by contrast tend to support a rise in individual costs to reduce climate change. A second dimension, basically perpendicular to the punishment-cost dimension and going diagonally from lower left to upper right, contrasts behaviors that emphasize political strategies (support for the Paris Agreement, support for subsidies) with behaviors that emphasize individual activities (reduce one’s energy use, discuss climate change with others). German, United Kingdom, and Norwegian respondents tend toward political strategies, whereas French respondents are more inclined to support individual strategies. The same caveat applies to behavioral tendencies as to emotions and appraisals, namely is, although the correspondence analysis pins down credible relative differences between nations, the overall effect of countries in accounting for differences in behaviors is small (𝜂^2^ = 0.04).

### 3.3. Within-country differences

It can be argued that comparing countries at an aggregated level conceals substantial differences within countries; for example, political and ideological variance might be larger within than between countries. To examine this possibility, we created subgroups within countries. Specifically, we performed a dichotomous split of each national sample according to four variables: age, gender, education, and political orientation (see Supplementary Table S2 in Supplementary material).

Age, measured with six ordered categories of 10 years, was dichotomized by merging the lower three categories that include all participants up to an age of 44 years (*n*_young_ = 1766), and merging the upper three categories with participants older than 44 years (*n*_old_ = 2,282). Gender was dichotomized in males (*n*_male_ = 2032) and females (*n*_female_ = 2016). Education was dichotomized by contrasting participants with and without a university degree (*n*_university_ = 1,229, *n*_no-university_ = 2,819). Political orientation was dichotomized at the median of 5 on an 11-point rating scale into a politically left leaning and a right leaning group (*n*_left_ = 2,529, *n*_right_ = 1,120); participants with a value of 5 were assigned to the ‘left’ group.

This dichotomization allows us to contrast across all countries differences between younger and older individuals, males and females, people with and without a university degree, and those respondents who rated themselves as being more conservative (‘right’) with those who rated themselves as being more progressive (‘left’).

The same methodological approach was applied as before, but instead of using aggregated values on the country level, here we used two sub-samples for each country, representing opposing groups with respect to age, gender, education, and political orientation. Means for the five emotions were computed for all sub-samples, converted to pseudo-contingencies, and submitted to correspondence analysis. Here we only report analyses of emotions; analyses of appraisals and behavioral tendencies yield very similar configurations of the country subgroups and can be found in the Supplementary material.

**Figure 4:**
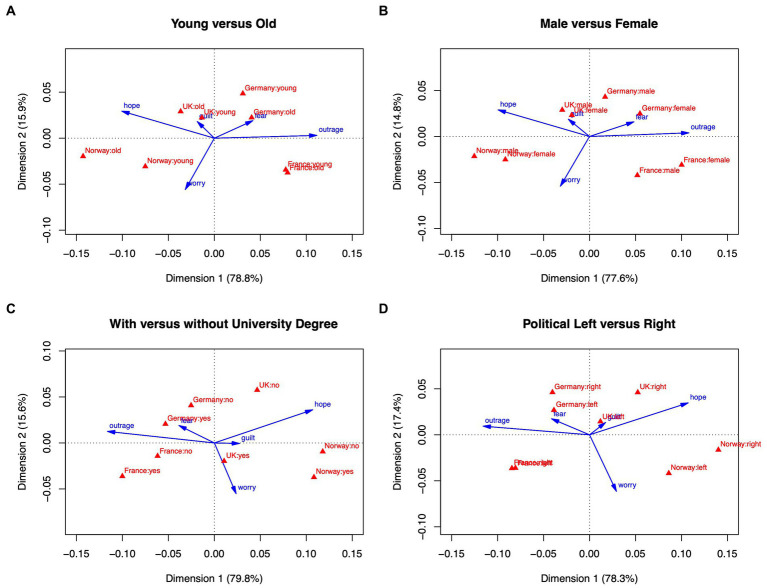
Correspondence analysis of emotions with dichotomized country background variables. **(A)** Young vs. old participants. **(B)** Males vs. females. **(C)** Participants with vs. without university degree. **(D)** Left vs. right political orientation. Emotions are depicted as blue vectors, country sub-samples as red triangles.

Figure 4 shows the two-dimensional correspondence analysis plots; emotions are depicted as vectors, and national sub-samples as points (triangles). All four analyses clearly show that within-country differences are generally smaller than between-country differences. The configurations of the countries remain unchanged compared to the aggregate analysis without within-country subgroups (Section 3.2, Figures 1–3). Figure 4A, contrasting younger and older people, shows that despite the emergence of a difference between older and younger Norwegians (older Norwegians being most hopeful), the order of the countries on the horizontal axis remains unchanged compared to the aggregate analysis, with France (old and young) expressing strongest outrage, followed by Germany and United Kingdom, and with Norway (old and young) expressing most hope. By and large, the same pattern emerges when contrasting males and females (Figure 4B), participants with and without a university degree (Figure 4C), and politically left-leaning with more right-leaning individuals (Figure 4D). This finding strongly suggests that with respect to emotional reactions to climate change, national differences dominate differences within nations, at least when focusing on sociodemographic attributes such as age or gender in the context of the five emotions investigated in this study.

### 3.4. Embedding climate emotions into cultural context

**Figure 5:**
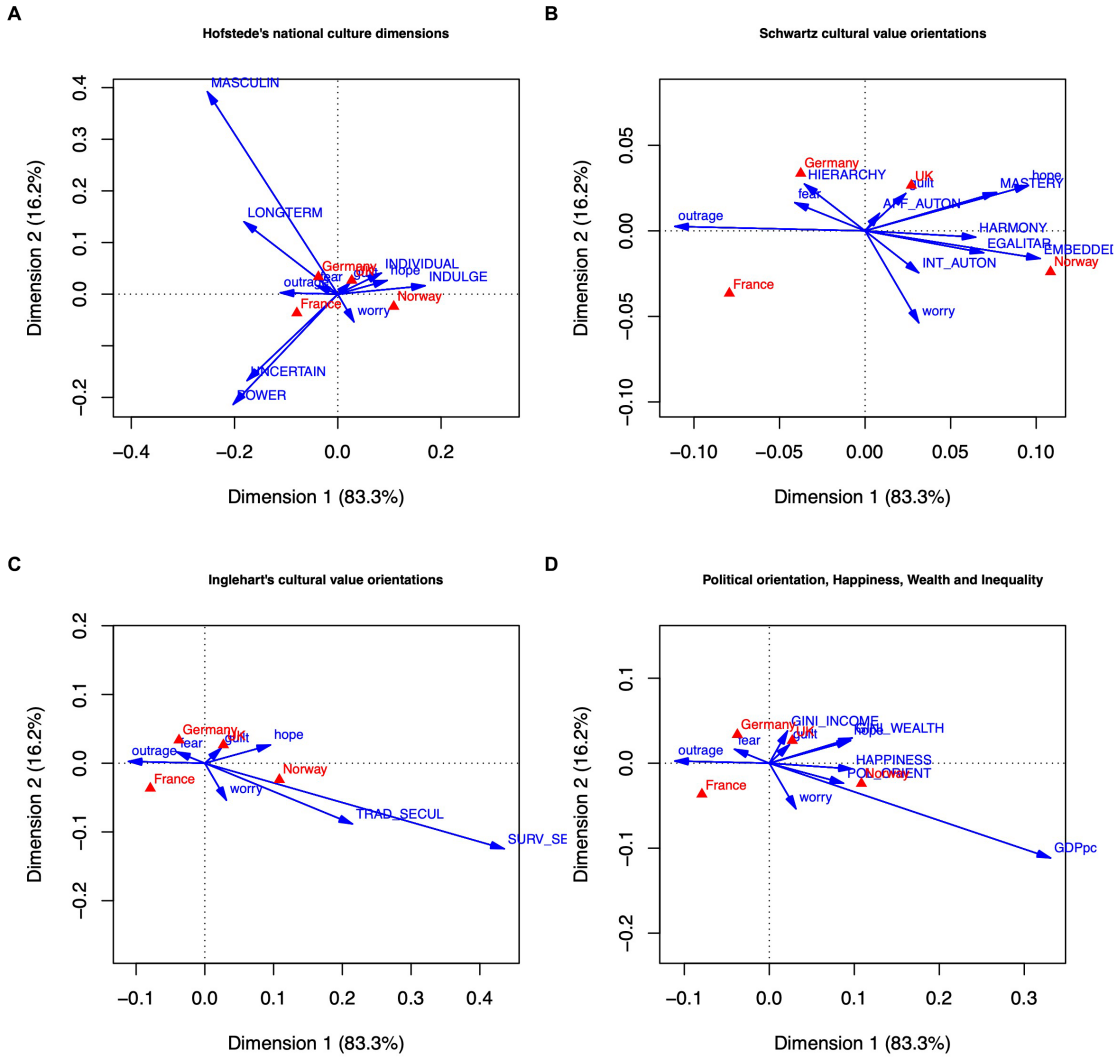
Correspondence analysis of emotion-country means with supplementary context variables included. **(A)** Hofstede’s national culture dimensions. **(B)** Schwartz’ cultural value orientations. **(C)** Inglehart’s cultural value orientations. **(D)** Political orientation, happiness, wealth and inequality. Emotions and supplementary variables are depicted as blue vectors, countries as red triangles; supplementary variables are in capital letters.

To support interpretation of national differences in emotional responses, appraisals, and behavioral tendencies concerning climate change we add a collection of pertinent cultural context variables to the findings depicted in Figures 1–3. We chose three well-known theoretical models for which relevant data are available publicly (Figure 5):

(a) Hofstede’s six dimensions of cultural values (Hofstede, 2001); aggregated data for France, Germany, Norway, and the United Kingdom were retrieved from Hofstede (2023). The masculinity versus femininity dimension represents a preference for materialistic and achievement values versus a preference for cooperation and tenderness, respectively. The power distance dimension contrasts acceptance of power hierarchies with a preference for equality of power. The individualism versus collectivism dimension contrasts individual and non-social societies with societies that consider group loyalty as more important. The uncertainty avoidance dimension discriminates societies that are rigid and orthodox in handling the future from societies with more relaxed and unorthodox practices. The long-term orientation is a dimension contrasting flexibility and change with maintaining traditions and norms. Indulgence versus restraint is a dimension representing the contrast between hedonistic and suppressive societies concerning human drives and needs.

(b) Schwartz seven cultural value orientations as described in Schwartz (2006); data were retrieved from Schwartz (2008). The seven orientations can be grouped into three superordinate dimensions as follows. Autonomy versus embeddedness contrasts an emphasis on the autonomous individual with the view of individuals as being essentially part of a social group. Autonomy can be divided into intellectual and affective autonomy. Egalitarianism versus hierarchy represents the difference between societies that view people as essentially equal and societies that rely on hierarchical structures. The third dimension represents a distinction between cultures that try to live in harmony with their social and natural environment and cultures that strive for control and mastery of the environment. Conceptual similarities with Hofstede’s dimensions are apparent, but will not be discussed in this article.

(c) Inglehart’s two cultural dimensions (Inglehart and Baker, 2000); data were retrieved from Inglehart and Welzel (2023). Ingelhart and Welzel’s cultural map positions nations in a two-dimensional system with orthogonal dimensions. The first dimension contrasts traditional versus secular-rational values; traditional values emphasize family and religion, whereas secular values de-emphasize tradition and accept practices such as abortion and divorce. The second dimension contrasts survival values versus self-expression values; survival values imply a strong inclination for economic security and an ethnocentric attitude, whereas self-expression values imply tolerance, participation, and environmental protection. Kaasa (2021) provides a thorough comparison and integration of Hofstede’s, Schwartz’, and Inglehart’s models.

In addition, we computed from our own data the average political orientation (see Section 2.3) for each country. The original 11-point rating scale was rescaled to five levels to make it comparable to the other dimensions in the current analysis; 1 indicates a left and 5 a right political orientation. Further, we added two economic variables, the GDP (Gross Domestic Product in Dollars) *per capita* (pc) (Wikipedia, 2023a) as an aggregated indicator of wealth, and the GINI indices of income and wealth inequality as indicators of a country’s economic inequality (Wikipedia, 2023b). The GINI index is a normalized coefficient with zero indicating extreme equality and 1 indicating extreme inequality in the distribution of income or wealth. Finally, we also added an aggregate measure of life satisfaction taken from the World Happiness Report 2023 (Helliwell et al., 2022), with 0 indicating the worst and 10 indicating the best possible satisfaction with life. Supplementary Table S3 in Supplementary material summarizes the context variables.

The values of all context variables were converted to pseudo-contingencies (see Section 2.4) and analyzed via correspondence analysis. We kept the configurations that were obtained for emotions, appraisals, and behavioral tendencies (Figures 1–3) as fixed, and added the cultural context scales as supplementary variables (Greenacre, 2016). That is, the context scales were fitted into the original configuration without changing it. The interpretation proceeds as in the previous correspondence analyses. That is, projections of the country points onto the vectors of the supplementary scales represent the position of the country on the scale.

Results of embedding the supplementary context variables into the emotion configuration are depicted in Figure 5; supplementary variables are printed in capital letters. Figure 5A includes Hofstede’s six dimensions. Masculinity and long-term orientation point more or less in the same direction toward the upper left, suggesting that the expression in countries such as Germany and France of relatively high amounts of fear and outrage is associated with more masculinity and long-term orientation compared to the United Kingdom and Norway. Pointing toward the lower left are uncertainty avoidance and power distance, suggesting that respondents in France would tend to accept strict rules and power hierarchies, and that this is somewhat associated with expressing more worry. Virtually parallel to the horizontal axis and the hope vector are the individualism and indulgence dimensions, indicating that in Norway expressing more hope with respect to climate change is also associated with a stronger preference for individualistic-hedonic gratifications.

Figure 5B incorporates Schwartz’ cultural dimensions into the emotion configuration. The horizontal axis, representing a continuum from outrage to hope, is also closely associated with cultural scales such as harmony, egalitarianism, embeddedness and intellectual autonomy, and to a lesser extent mastery, all increasing with hope. The hierarchy dimension, on the other hand, is closely associated with increasing fear and outrage. This suggests that the populace of countries such as France and Germany that rely on hierarchical structures tend to experience stronger fear and outrage concerning climate change. Conversely, Norway is a country characterized by reliance on social harmony egalitarianism, and embeddedness, but also by an emphasis on mastery, which is closely associated with hope.

Figure 5C includes Inglehart’s two cultural value dimensions. With respect to the four countries France, Germany, Norway, and the United Kingdom, both dimensions basically point in the same direction, with Norway being located toward the secular/self-expression pole, and France and Germany showing relatively more reliance on tradition and survival values. Both cultural values are also correlated with the horizontal axis, suggesting that higher degrees of self-expression and secularity are associated with more hope, but also with more worry, concerning climate change. In contrast, more reliance on tradition and survival is associated with more fear and outrage.

Figure 5D adds economic variables, political orientation, and the happiness index to the emotion configuration. Not surprisingly, GDPpc representing the wealth of a nation is closely associated with the contextual variables happiness and conservative political orientation; these three variables go together with the emotion hope. Norway scores high on that dimension. Economic inequality as represented by the Gini-indices for income and wealth is largely orthogonal to GDP and associated with guilt; Germany and United Kingdom respondents score relatively high on inequality. France, on the other hand, is characterized by a more left political orientation, by a relatively low GDP, and less inequality, which is associated with outrage and worry expressed by respondents with respect to climate change.

To summarize, the basic finding of an emotion dimension going from outrage to hope, can be supplemented with contextual variables such as individualism, hedonism, egalitarianism, happiness and wealth, all associated with more hope concerning climate change. In contrast, cultural values such as hierarchy, tradition, long-term orientation and economic inequality are associated with more fear and outrage concerning climate change. Norway typically represents the hope/egalitarian/wealthy end, whereas France and Germany rather represent the outrage/hierarchical/inequality end. It should be stressed that these data do not allow for causal interpretations of any kind. It seems plausible to speculate that, for example, inequality in the distribution of wealth leads to outrage, and climate change is attributed to those who are high in the power hierarchy. By contrast, a wealthy country such as Norway may rely more on flexible technical solutions of climate change, and thus express more hope. However, the panels in Figure 5 are purely descriptive, with the main purpose of providing a background of cultural and economic dimensions to embed the climate change related emotions in a larger context. For simplicity, we focus in the manuscript on the cultural context with respect to emotions and relegate the results concerning appraisals and behavioral tendencies to Supplementary material (Supplementary Figures S3, S4).

## 4. Discussion

In this study, we examined relationships between appraisals of climate change and emotions and between emotions and behaviors targeted toward climate change. Furthermore, we explored differences between respondents from four countries (France, Germany, Norway, United Kingdom) concerning emotional responses in the face of climate change while also investigating related country differences in climate appraisals and behaviors. Our data are a subset from an international survey on European Perceptions of Climate Change (EPCC: Steentjes et al., 2017).

The first research question, approached by using canonical correlation analysis techniques, draws on appraisal-theoretic frameworks and attempts to identify regularities in appraisal-emotion and emotion-behavior linkages (Section 3.1). The second research question, approached with correspondence analysis techniques, focuses on national differences, with the analyses revealing typical patterns of emotional responses, appraisals, and behaviors across countries (Section 3.2). We used large representative samples from the international study comprising about 1,000 respondents from each of the four countries, and we assessed three sets of measures, namely, five emotions, ten appraisal scales, and seven behavioral options (see Table 1). The entire study included many more items (Steentjes et al., 2017); here we selected those measures that tapped into the concepts which are relevant for the analysis of emotional responses toward climate change.

Our first analytic focus was on relations between appraisals, emotions, and behavioral intentions, as postulated by appraisal-theoretic models (Frijda et al., 1989; Lerner and Keltner, 2000). The first analysis relates the set of appraisals with the set of emotions via canonical correlation analysis (Thompson, 1991). We found that two canonical correlations are substantial, accounting for 54% of the variance. The first canonical correlation relates appraisals of human versus natural causation and moral concern with the negative emotions worry, fear, and outrage; the second canonical correlation relates appraisals of efficacy and technological solvability to feelings of increased hope and reduced outrage. While we cannot draw causal conclusions from these results, they are compatible with assuming that appraisals which indicate personal involvement (e.g., moral concern) trigger negative emotions such as worry and outrage (but not guilt). Furthermore, appraisals of human agency (e.g., perceived collective efficacy) elicit hope and additionally inhibit outrage, leading to a calmer and more hopeful emotional response.

The second analysis examined associations between emotions and behavioral intentions to mitigate climate change. The first canonical correlation associates emotions of worry and outrage with behavioral tendencies to reduce one’s energy consumption, to discuss climate change with others, or to challenge other people about climate change engagement. The second canonical correlation associates emotions of hope, guilt, and reduced outrage with support for policies to raise prices and taxes on harmful energy consumption, but with a lower tendency to discuss climate change with others or to challenge other people about climate change. The emotional bundles constituting the first and second emotional canonical variant in the emotion-behavior analysis are the same as those found in the analysis of appraisal-emotion relationships.

Thus, in appraisal-theoretic terms and simplifying our findings to some degree, we can speculate about two emotional pathways. In one pathway, appraisals of personal involvement elicit negative emotions, especially worry and outrage, which in turn trigger individual behavioral strategies to deal with climate change (e.g., to reduce energy consumption) (see Tables 4, 5, 8, 9, first canonical correlation). In a second pathway, appraisals of human agency elicit feelings of hope and calmness, which in turn trigger behavioral strategies to support increases in prices and taxes on harmful energies (see Tables 4, 5, 8, 9, second canonical correlation). Differences between country samples are reflected in the respective canonical covariates (see Table 6): French respondents yield highest scores on the appraisal of personal involvement as well as on negative emotions (worry and outrage). Norwegian respondents score highest on the human agency appraisal as well as on hope and calmness emotions. Respondents in France score highest on self-declared individual actions such as energy reduction, and those in Norway present highest scores on support for behaviors involving increases of energy costs.

Our second research focus is on the relationship between emotions and national context, highlighting national differences in emotional responses to climate change (as well as national differences in appraisals and climate related behaviors). Applying correspondence analysis (Greenacre, 2016) to emotional responses across the four countries yields a distinct pattern mapping the four countries onto a main dimension of outrage versus hope (Figure 1). That is, the five emotion ratings (worry, outrage, fear, hope, guilt) can be reduced to one dimension (accounting for 83% of the variance) contrasting feelings of outrage and fear with feelings of hope. On that dimension, we find France located at the ‘outrage’ end, and Norway located at the ‘hope’ end; Germany is leaning more toward outrage and fear, and the United Kingdom is slightly leaning toward hope, yet also toward feeling guilt. Perpendicular to the outrage-hope dimension emerges a dimension mainly characterized by feelings of worry, with respondents in France and Norway feeling relatively more worry and the United Kingdom and Germany relatively less worry; however, this second dimension discriminates national emotion patterns less than the first dimension (it accounts for only 16% of the variance).

The contrast seen here between outrage and hope is plausible and compatible with theoretical assumptions, and it also parallels the findings from the canonical correlation analyses. For example, outrage can be considered a deontological emotion (Böhm and Pfister, 2000), elicited by immoral actions of individuals or organizations who then become the target of outrage. Conversely, hope can be classified as a consequentialist emotion (Böhm and Pfister, 2000), elicited by anticipating future consequences that are evaluated as positive and pleasant. Depending on a person’s attentional focus, outrage or hope can dominate the emotional response. Hence, French respondents seem to concentrate mainly on the culprits of climate change (e.g., industries, politicians), whereas Norwegian respondents pay more attention to positive consequences that could be attained when dealing with climate change. The hope expressed by Norwegian respondents may partly derive from the relatively high levels of technology optimism (and familiarity) seen in the country, related for example to electric vehicles (Arnold et al., 2016) and carbon capture and storage (Merk et al., 2022). The relatively high levels of hope reported in Norway are also in line with previous findings, as are the higher levels of generalized trust found in the country (Arnold et al., 2016). By contrast, French public opinion on renewable energy is less optimistic (Arnold et al., 2016).

Though the focus of this paper is on emotions, we conducted analogous correspondence analyses with respect to appraisals and behaviors. Relating ten appraisal scales with responses observed in the four countries yields two dimensions on which the country samples show distinct differences. One dimension (accounting for 63% of variance) contrasts judgments of personal involvement with judgments of geographical closeness. Personal involvement comprises a bundle of appraisal scales all pointing in the same direction: social closeness, perceived collective efficacy, and moral concern indicate that people focus on questions such as ‘am I affected?’, ‘can we do something?’, ‘am I morally obligated to do something?’. Positive responses to these questions indicate increased personal involvement in climate change issues; French and Norwegian respondents score high on this pole of the dimension. The opposite pole is marked by geographical closeness, that is, people ask ‘are places here rather than regions geographically far away from where I live affected by climate change?’. Maybe unexpectedly, a judgment that the effects of climate change will be ‘close to where I live’ seems to operate against personal and moral involvement; maybe it constitutes a more sober and cool-hearted judgment of being affected or not, or it could indicate an attitude of denial. It is interesting to see that the three dimensions of psychological distance that we included in our measures are not all closely correlated. German respondents and, slightly less, United Kingdom respondents score relatively high on the geographical ‘close’ end of this appraisal dimension.

A second, perpendicular dimension emerges contrasting judgments of technological solvability with judgments of human causation and negative impacts of climate change. The location of German and French respondents on this dimension indicates that they see climate change as having negative impacts and being caused by humans. Respondents from Norway and the United Kingdom, in contrast, tend more toward the technological end, that is, toward believing that climate change can be successfully dealt with by relying on science and technology. It seems plausible that a confidence in technological solutions counteracts anticipating negative impacts of climate change; also, it is conceivable though somewhat speculative that in Norway, a country whose wealth is based on oil technology and hydroelectric power (Arnold et al., 2016), respondents tend to judge technological solutions as desirable and feasible. Similarly, United Kingdom climate mitigation policy tends to prioritize technological solutions over behavior changes and citizens’ personal responsibility (Environment and Climate Change Committee, 2022; Skidmore, 2022). On the other hand, the German population has a reputation of being ‘techno-skeptical’, emphasizing the negative effects of many modern technologies, notwithstanding the fact that Germany’s wealth is largely based on traditional industries considered as contributing to climate change.

Regarding behavioral responses to climate change, we find one dimension contrasting behaviors which are directed at punishing others for not complying with climate change policies (here, the Paris Agreement), with behaviors representing strategies that increase individuals’ costs for energy consumption. Germany and France are located on the political punishment end, whereas United Kingdom and Norway lean toward the individual cost end of that dimension. A second dimension constitutes a contrast between political strategies, such as public subsidies, with individual strategies, such as trying to reduce one’s individual energy consumption. Respondents in Germany, the United Kingdom, and Norway tend more toward political strategies, whereas French respondents tend more toward individual strategies.

A joint examination of the three analyses shows interesting patterns. Respondents from France and Germany show corresponding patterns of emotions, appraisals, and behaviors: They show high outrage and fear; high judgments of negative impact and human causation; and a tendency to support punitive rather than monetary (cost increase) strategies. A second corresponding pair of countries is Norway and the United Kingdom, whose samples show relatively strong feelings of hope (and guilt), consider technological solutions as feasible, and would approve of increasing prices and taxes for harmful energy use rather than punishing violators of the Paris Agreement. However, these pairs are not aligned on all dimensions; for example, French respondents support individual behaviors such as reducing one’s energy consumption, whereas German respondents favor political strategies such as the provision of subsidies.

One might object to comparing countries as single and homogenous entities, arguing that within-country differences might be larger than between-country differences; for example, gender differences or age might account for more variance in attitudes and emotions than national differences. We used four sociodemographic background variables – gender, age, education, and political orientation – to check this objection. It turns out that subsamples contrasting these background variables are consistently more similar than samples of different countries. This suggests that at least with respect to the responses to climate change included in this study, national characteristics dominate attributes such as age or political orientation.

It can be assumed that there are more underlying differences in cultural norms and values that regulate which emotions (and appraisals and behaviors) are considered appropriate with regard to climate change. Socio-cultural research has long aimed to establish general dimensions along which countries differ with respect to fundamental norms and values. We included three pertinent models of cultural values to assist the interpretation of national differences: Hofstede’s model of cultural dimensions (Hofstede, 2001), Schwartz’ model of cultural value orientations (Schwartz, 2006), and Inglehart’s model of value orientations (Inglehart and Welzel, 2023). It turns out that national peculiarities and differences as inferred from the basic analysis of emotional responses shown in France, Germany, Norway, and the United Kingdom, are by and large compatible with the relative positions of the four countries on those cultural dimensions. Norwegians consistently consider secular values such autonomy, egalitarianism, and mastery as important, whereas Germany and France place more importance on traditional values such as social hierarchies, masculinity, and uncertainty avoidance (see Figure 5 for an overview). It is conceivable that persons in a country such as Norway will generally express more hope regarding the future than those in countries such as Germany and France where values of economic survival and uncertainty avoidance play a more prominent role, eliciting outrage rather than hope when thinking about causes and consequences of climate change. Note that these national differences are relative to the four countries studied; in a world-wide context all four European countries would be highly similar on all dimensions if compared to African or East Asian countries (Inglehart and Welzel, 2023). It may be argued that on an even more fundamental level a country’s economic conditions underpin the prevalent values, norms, and emotions. We contrasted France, Germany, Norway, and the United Kingdom on economic characteristics such as Gross Domestic product and inequality of wealth and income (Figure 5D). Not surprisingly, Norway stands out as the wealthiest country in terms of gross domestic product *per capita*, which might be the simplest and most parsimonious explanation for respondents’ more hopeful view concerning climate change; in contrast, respondents from Germany and France, countries with relatively lower individual wealth, tend to express a more fearful and angry view. Inequalities in income and wealth account for noticeably smaller national differences (Figure 5D), perhaps because people tend to compare themselves to similar others so that they may be less aware of these inequalities in their everyday lives.

Some limitations of this study need to be emphasized. Concerning generalizability of our findings, it should be noted that due to the large and representative samples, we may reliably generalize to the underlying population of individuals. However, the collection of measures used as well as the four countries studied are quite selective and partly arbitrary. The selection of countries was driven mainly by organizational and practical factors, and is not based on theoretical considerations. Generalizations to other countries hardly seem feasible, though similar results might be expected in countries of comparable economic and social composition (for a characterization of the four countries with respect to their socio-cultural profiles related to climate change, see Arnold et al., 2016). More importantly, the five emotions measured are merely a small subset of possibly relevant emotions concerning climate change; in particular, discrete emotions such as despair, sadness, regret, or shame, that have been treated as relevant for climate change research, were not assessed. In fact, it is quite likely that the emotional response to climate change is more complex than can be inferred from the five emotions examined in this study. Furthermore, although we generally rely on an appraisal-theoretic background concerning the role of emotions, all analyses presented are exploratory and descriptive. The existing knowledge on climate change emotions and the nature of the data available do not allow us to formulate more precise statistical hypotheses, in particular, no causal hypotheses can be tested. However, our large and representative samples enable reliable and robust correlational analyses; this, in turn, provides a solid starting point for more specific and causal interpretations. Of course, further research, for example employing controlled experiments, more specific measures, and a broader range of pertinent emotions is needed to further scrutinize the role of emotions in people’s responses to climate change. One route this future work could take is to investigate the temporal dynamics of climate emotions. For example, emotions that have resulted from a specific appraisal of the situation can in turn focus attention to specific new information, guiding information processing and consequently influencing subsequent appraisals (Pfister and Böhm, 2008). Alternatively, attempts to cope with negative emotional reactions to the threats of climate change may attenuate the emotional response and thereby reduce the motivation to act (Ogunbode et al., 2019). We have not been able to address these topics with our data, but we see them as promising and needed avenues for future research.

## Data availability statement

The datasets presented in this study can be found in online repositories. Data supporting this study are openly available from the United Kingdom Data Service at https://doi.org/10.5255/UKDA-SN-8325-1 (Study number: 8325).

## Ethics statement

The studies involving human participants were reviewed and approved by Research Ethics Committee of the School of Psychology at Cardiff University. The patients/participants provided their written informed consent to participate in this study.

## Author contributions

GB, RB, CM, KS, MS, NP, WP, and ET collaboratively designed the study and developed the questionnaire. GB and H-RP performed the statistical analyses and wrote the first draft of the manuscript. All authors contributed to the article and approved the submitted version.

## Funding

This research was conducted as part of the “European Perceptions of Climate Change” project, which was funded by the Joint Programme Initiative on Climate Change (JPI-Climate) with associated grants from Cardiff University Sustainable Places Research Institute, School of Psychology and the Economic & Social Research Council, ESRC [grant number ES/M009505/1], France’s Agence Nationale de la Recherche [grant number ANR-14-JCLI-0003], the KLIMAFORSK programme of the Norwegian Research Council [NFR; project number 244904], and the German Federal Ministry of Education and Research [grant number 01UV1403]. The project received co-funding from the cooperation agreement between Equinor (formerly Statoil) and the University of Bergen [Akademiaavtale; project number 803589].

## Conflict of interest

The authors declare that the research was conducted in the absence of any commercial or financial relationships that could be construed as a potential conflict of interest.

## Publisher’s note

All claims expressed in this article are solely those of the authors and do not necessarily represent those of their affiliated organizations, or those of the publisher, the editors and the reviewers. Any product that may be evaluated in this article, or claim that may be made by its manufacturer, is not guaranteed or endorsed by the publisher.
