# Supplementary material for: Emotional reactions to climate change: a comparison across France, Germany, Norway, and the United Kingdom
Source: Front Psychol. 2023 Jul 6;14:1139133. doi: 10.3389/fpsyg.2023.1139133 (PMC10358841; doi:10.3389/fpsyg.2023.1139133)
Supplement: Supplementary file 1 [file Data_Sheet_1.pdf]

frontiers

Supplementary Material

Emotional reactions to climate change: a comparison across France, Germany, Norway, and the United Kingdom

Gisela Böhm*, Hans-Rüdiger Pfister, Rouven Doran, Charles A. Ogunbode, Wouter Poortinga, Endre Tvinnereim, Katharine Steentjes, Claire Mays, Raquel Bertoldo, Marco Sonnberger, Nicholas Pidgeon

*** Correspondence:** Gisela Böhm: gisela.boehm@uib.no

1 Supplementary Figures and Tables

1.1 Supplementary Tables

Table S1. Statistics for all variables by country. (a) Emotion ratings, (b) Appraisal ratings, (c) Behavior ratings. *n* = effective sample size, mean = arithmetic mean, sd = standard deviation, lcl = lower confidence limit, ucl = upper confidence limits (limits are for 95% confidence interval).

Country	Variable	*n*	mean	sd	lcl	ucl
(a) Emotion Ratings:						
France	worry	1,008	3.28	1.03	3.22	3.35
France	hope	996	2.30	1.08	2.24	2.37
France	fear	1,010	2.71	1.20	2.64	2.79
France	outrage	996	3.04	1.28	2.96	3.12
France	guilt	1,007	2.17	1.13	2.10	2.24
Germany	worry	990	2.99	1.09	2.93	3.06
Germany	hope	979	2.62	1.00	2.56	2.69
Germany	fear	989	2.78	1.13	2.71	2.85
Germany	outrage	982	2.90	1.19	2.83	2.97
Germany	guilt	977	2.38	1.05	2.31	2.45

Country	Variable	*n*	mean	sd	lcl	ucl
Norway	worry	1,002	3.01	0.97	2.95	3.07
Norway	hope	949	2.59	1.07	2.52	2.65
Norway	fear	987	2.11	1.07	2.04	2.17
Norway	outrage	974	1.93	1.09	1.86	2.00
Norway	guilt	978	2.04	1.00	1.97	2.10
UK	worry	1,020	2.69	1.11	2.62	2.76
UK	hope	1,006	2.49	1.13	2.42	2.56
UK	fear	1,016	2.30	1.20	2.22	2.37
UK	outrage	1,014	2.28	1.25	2.21	2.36
UK	guilt	1,014	2.03	1.12	1.96	2.10

(b) Appraisal Ratings:

France	causation	993	3.61	0.94	3.55	3.67
France	impact	986	3.91	0.86	3.85	3.96
France	tempDist	992	4.27	1.12	4.20	4.34
France	socDist	995	3.67	1.13	3.60	3.74
France	geoDist	995	1.96	1.12	1.89	2.03
France	injNorm	1,007	3.76	1.33	3.68	3.85
France	desNorm	989	3.10	1.23	3.03	3.18
France	efficacy	984	3.77	1.12	3.70	3.84
France	moralConcern	1,004	3.24	1.11	3.17	3.31
France	technoSolve	977	2.69	1.18	2.61	2.76
Germany	causation	933	3.59	0.96	3.52	3.65
Germany	impact	928	4.02	0.81	3.97	4.07
Germany	tempDist	970	4.14	1.31	4.06	4.22
Germany	socDist	916	3.30	1.15	3.22	3.37
Germany	geoDist	925	2.77	1.07	2.70	2.83
Germany	injNorm	970	3.12	1.27	3.04	3.20
Germany	desNorm	944	2.74	1.09	2.67	2.81
Germany	efficacy	917	3.29	1.03	3.22	3.35
Germany	moralConcern	957	2.79	1.04	2.72	2.86

Country	Variable	*n*	mean	sd	lcl	ucl
Germany	technoSolve	898	2.84	1.07	2.76	2.91
Norway	causation	993	3.29	0.75	3.24	3.33
Norway	impact	959	3.55	0.91	3.50	3.61
Norway	tempDist	972	4.17	1.25	4.10	4.25
Norway	socDist	985	3.63	1.18	3.56	3.70
Norway	geoDist	971	2.28	1.36	2.19	2.37
Norway	injNorm	973	3.62	1.34	3.54	3.71
Norway	desNorm	978	3.13	1.32	3.05	3.22
Norway	efficacy	992	3.57	1.36	3.49	3.66
Norway	moralConcern	987	3.03	1.23	2.95	3.11
Norway	technoSolve	972	3.32	1.23	3.24	3.40
UK	causation	986	3.39	0.93	3.33	3.45
UK	impact	977	3.56	0.94	3.51	3.62
UK	tempDist	992	4.16	1.28	4.08	4.24
UK	socDist	993	3.18	1.18	3.11	3.25
UK	geoDist	988	2.63	1.16	2.56	2.71
UK	injNorm	1,013	3.58	1.17	3.51	3.65
UK	desNorm	1,006	2.80	1.07	2.73	2.87
UK	efficacy	995	3.50	1.08	3.43	3.57
UK	moralConcern	1,011	3.07	1.30	2.99	3.15
UK	technoSolve	987	3.11	1.09	3.04	3.18

(c) Behavior Ratings:

France	needDiscuss	1,008	3.34	1.36	3.26	3.43
France	wouldChallenge	1,008	3.47	1.37	3.39	3.56
France	reduceEnergy	1,007	4.03	1.06	3.97	4.10
France	supportParis	969	4.11	1.01	4.04	4.17
France	punishViolators	971	3.97	1.11	3.90	4.04
France	policy_1publSubs	1,000	3.70	0.94	3.65	3.76
France	policy_2indiCost	1,003	2.06	1.01	1.99	2.12
Germany	needDiscuss	989	2.90	1.23	2.82	2.97
Germany	wouldChallenge	980	2.81	1.24	2.73	2.89

Country	Variable	*n*	mean	sd	lcl	ucl
Germany	reduceEnergy	983	3.42	1.12	3.35	3.49
Germany	supportParis	954	3.99	0.98	3.92	4.05
Germany	punishViolators	945	3.73	1.07	3.66	3.80
Germany	policy_1publSubs	977	3.49	0.96	3.43	3.55
Germany	policy_2indiCost	967	2.13	1.01	2.06	2.19
Norway	needDiscuss	996	3.20	1.41	3.11	3.29
Norway	wouldChallenge	987	3.38	1.42	3.29	3.46
Norway	reduceEnergy	982	3.69	1.33	3.60	3.77
Norway	supportParis	979	4.32	1.09	4.25	4.39
Norway	punishViolators	951	3.40	1.33	3.32	3.49
Norway	policy_1publSubs	975	3.78	0.93	3.72	3.84
Norway	policy_2indiCost	984	2.66	1.26	2.58	2.74
UK	needDiscuss	1,016	3.00	1.17	2.93	3.07
UK	wouldChallenge	1,014	3.25	1.24	3.18	3.33
UK	reduceEnergy	1,017	3.51	1.05	3.45	3.58
UK	supportParis	1,008	3.95	1.00	3.89	4.01
UK	punishViolators	1,006	3.58	1.09	3.52	3.65
UK	policy_1publSubs	1,009	3.62	0.95	3.56	3.68
UK	policy_2indiCost	1,010	2.46	1.05	2.39	2.52

Table S2. Dichotomized values for age, gender, education, and political orientation.

Variable	low	high
Age	young ≤ 44<br>(N = 1766)	old ≥ 45<br>(N = 2282)
Gender	male<br>(N = 2032)	female<br>(N = 2016)
Education	no university degree<br>(N = 2819)	has university degree<br>(N = 1229)
Political orientation	left<br>(N = 2529)	right<br>(N = 1120)

Note. Age was dichotomized by combining the lower and the upper three categories of a six-category scale, yielding a split at 44 years. Gender was dichotomized by distinguishing males and females. Education was dichotomized by contrasting participants with and without a university degree. Political orientation was dichotomized at the median of 5 on an 11-point rating scale; participants with a value of 5 were assigned to the 'low / left' group.

Table S3. Aggregated values for supplementary contextual variables.

Variable	France	Germany	Norway	UK
Political Orientation (1 .. 5)	2.77	2.63	2.88	2.75
Happiness (0 .. 10)	6.66	6.89	7.32	6.80
GDPpc (in $)	45.188	51.238	89.024	47.329
Gini-Index Income (0 .. 1)	0.32	0.32	0.28	0.35
Gini-Index Wealth (0 .. 1)	0.70	0.82	0.80	0.75
Hofstede Cultural Dimensions:				
(0 .. 100)				
Power Distance	68.00	35.00	31.00	35.00
Individualism	71.00	67.00	69.00	89.00
Masculinity	43.00	66.00	8.00	66.00
Uncertainty Avoidance	86.00	65.00	50.00	35.00
Long-Term Orientation	63.00	83.00	35.00	51.00
Indulgence	48.00	40.00	55.00	69.00
Inglehart Value Orientations:				
(Factor scores)				
Survival vs. Self-Expression	1.00	0.70	2.10	1.60
Traditional vs. Secular	0.60	1.10	1.20	0.20
Schwartz Cultural Values:				
(1 .. 7; item means)				
Harmony	4.21	4.54	4.40	3.91
Embeddedness	3.20	3.09	3.45	3.34
Hierarchy	2.21	1.82	1.49	2.33
Mastery	3.72	3.93	3.85	4.01
Affective Autonomy	4.39	4.20	3.69	4.26
Intellectual Autonomy	5.13	4.84	4.68	4.62
Egalitarianism	5.05	5.01	5.12	4.92

Note. See manuscript text for details about the variables.

1.2 Supplementary Figures

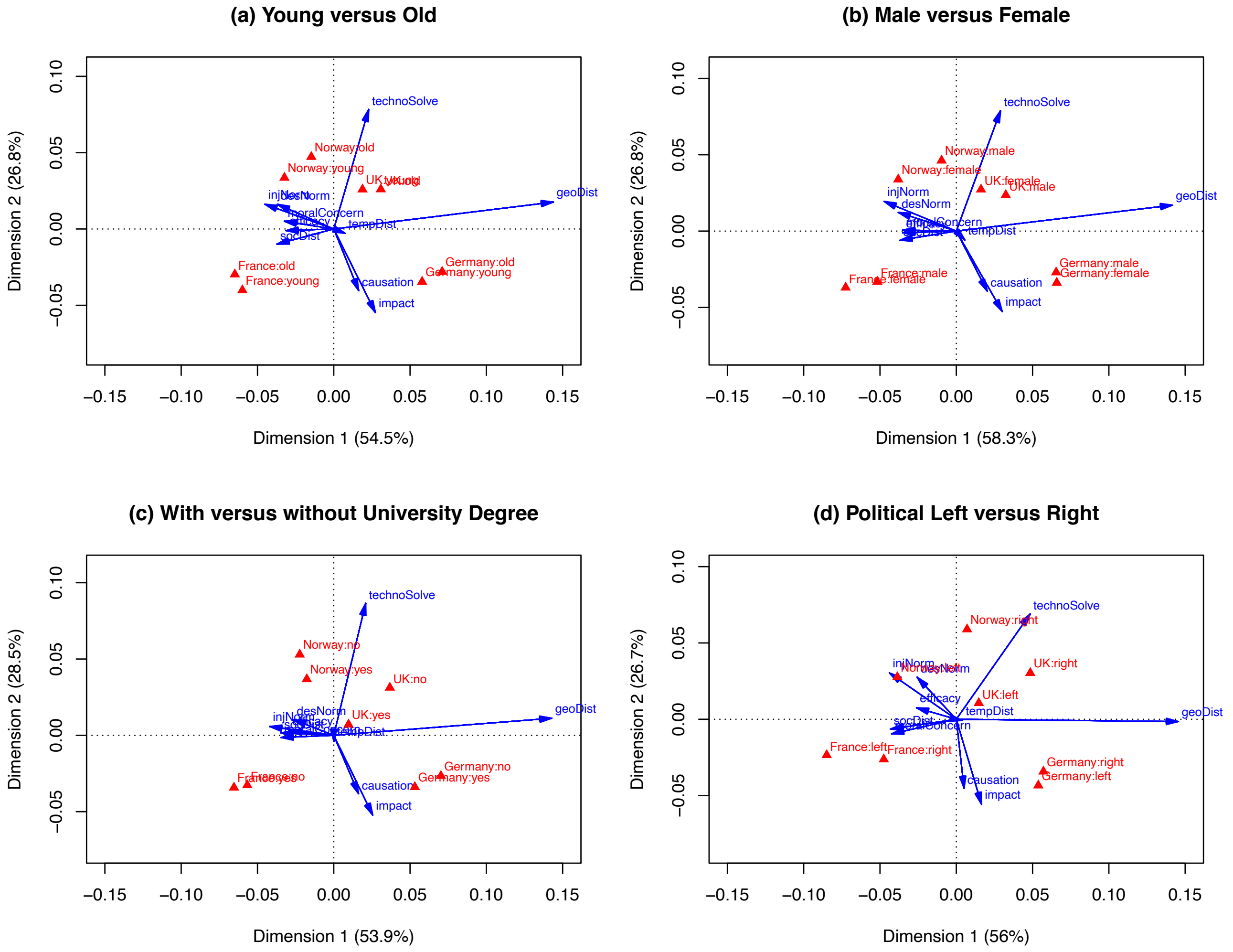

Supplementary Figure S1. Correspondence analysis of appraisals with dichotomized country background variables. (a) Young vs. old participants. (b) Males vs. Females. (c) Participants with vs. without university degree. (d) Left vs. right political orientation. Emotions are depicted as blue vectors, country sub-samples as red triangles.

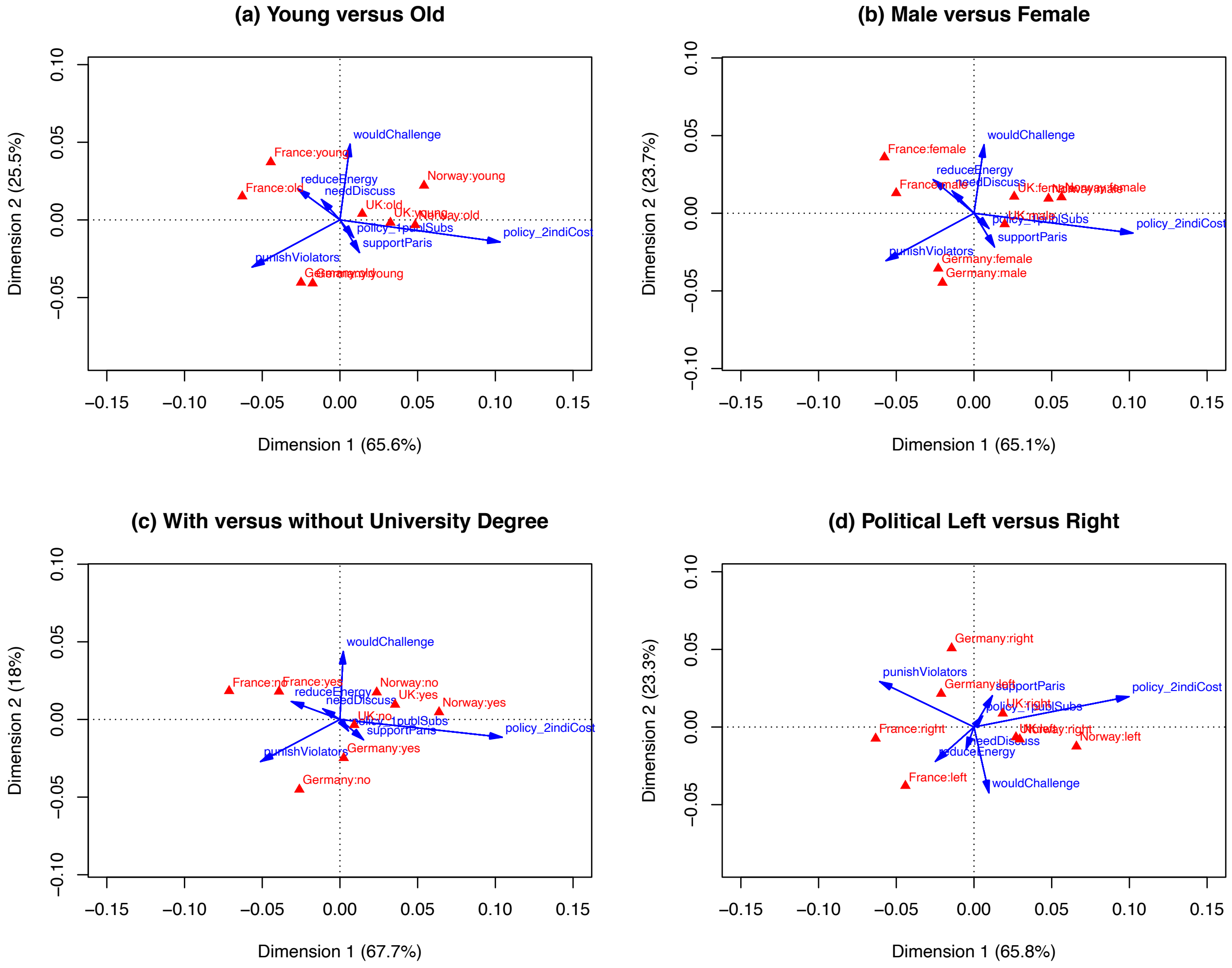

Supplementary Figure S2. Correspondence analysis of behaviors with dichotomized country background variables. (a) Young vs. old participants. (b) Males vs. Females. (c) Participants with vs. without university degree. (d) Left vs. right political orientation. Emotions are depicted as blue vectors, country sub-samples as red triangles.

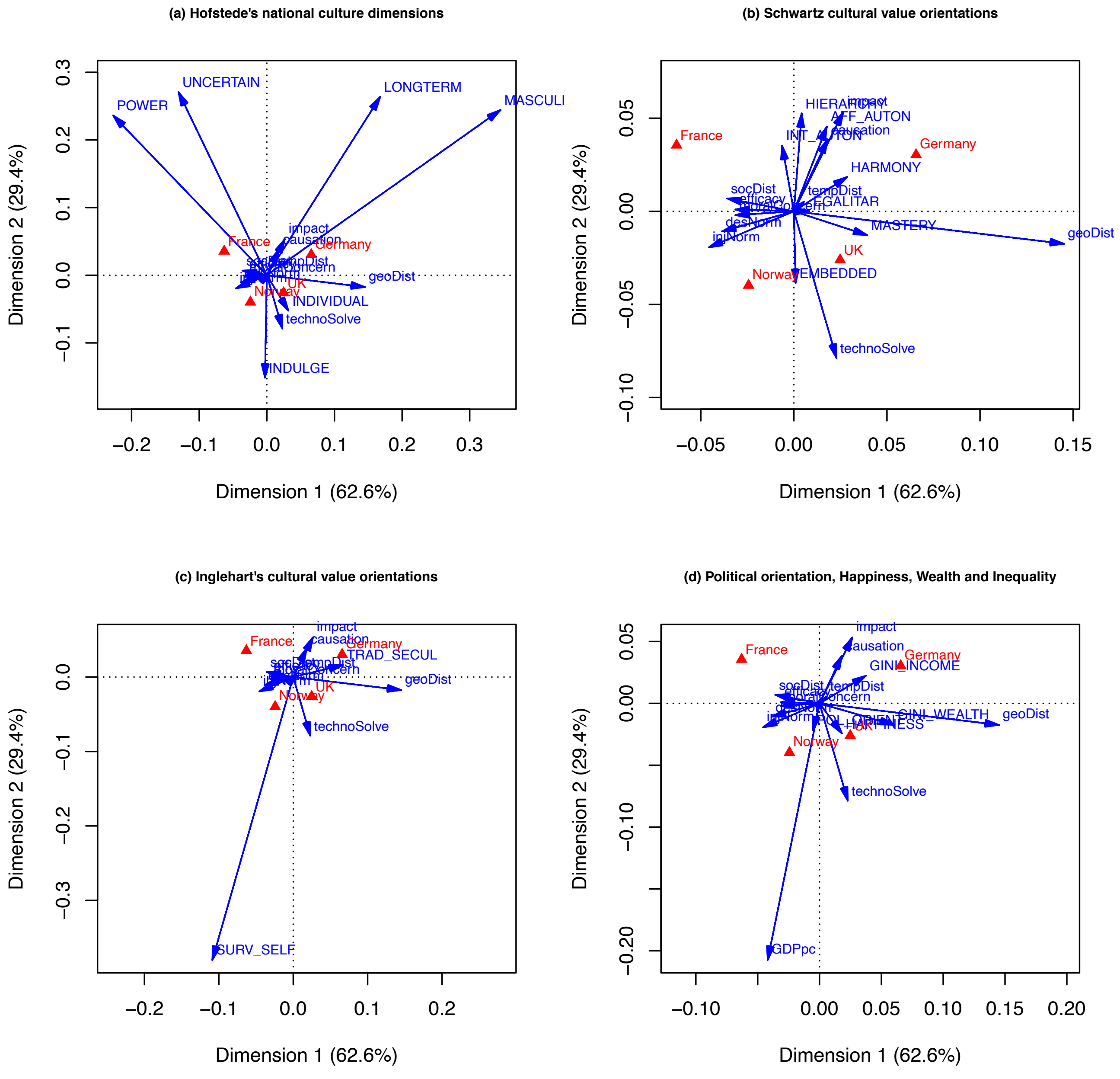

Supplementary Figure S3. Correspondence analysis of appraisal-country means with supplementary context variables included. (a) Hofstede's national culture dimensions. (b) Schwartz' cultural value orientations. (c) Inglehart's cultural value orientations. (d) Political orientation, happiness, wealth, and inequality. Emotions and supplementary variables are depicted as blue vectors, countries as red triangles; supplementary variables are in capital letters.

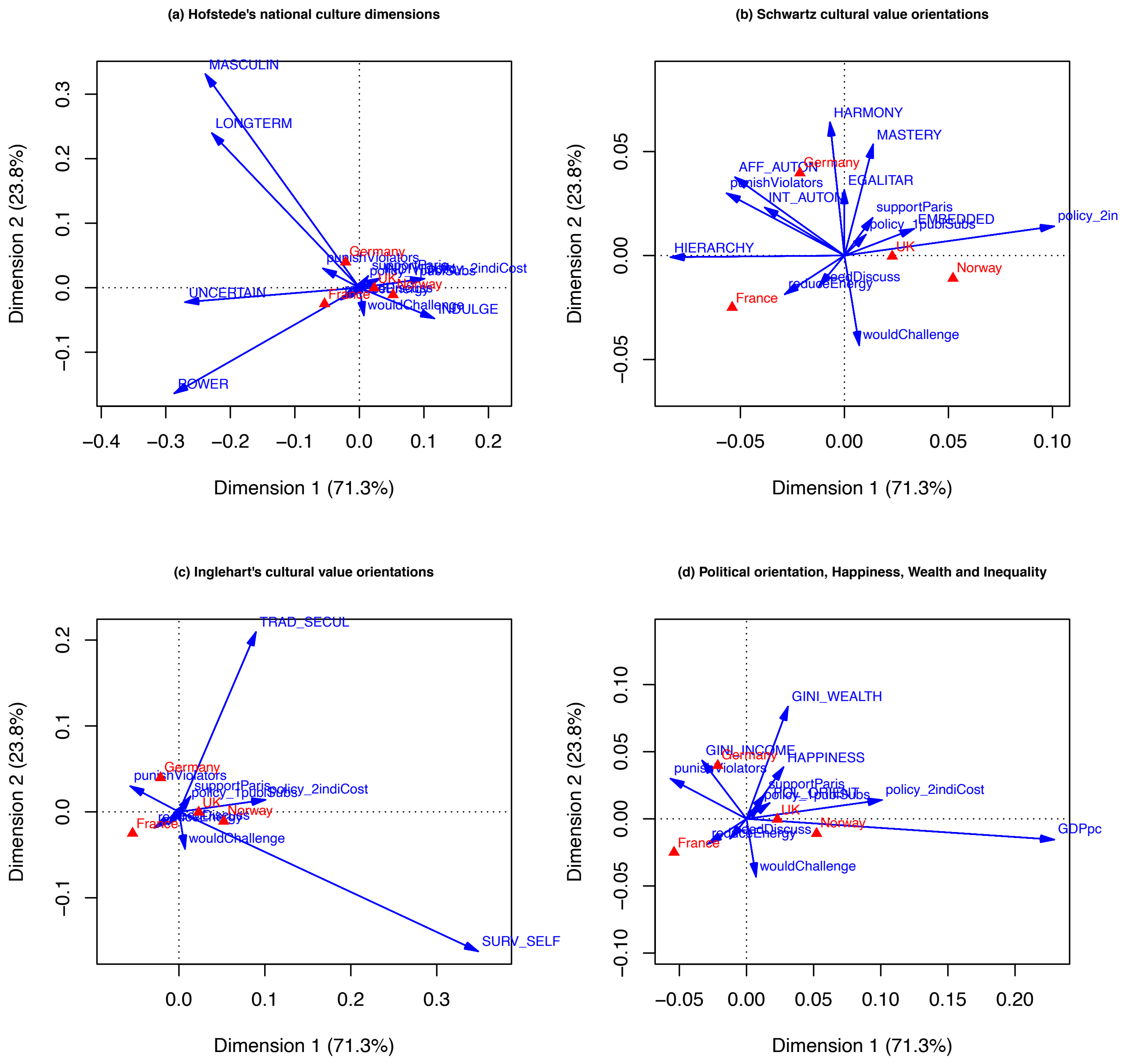

Supplementary Figure S4. Correspondence analysis of behavior-country means with supplementary context variables included. (a) Hofstede's national culture dimensions. (b) Schwartz' cultural value orientations. (c) Inglehart's cultural value orientations. (d) Political orientation, happiness, wealth, and inequality. Emotions and supplementary variables are depicted as blue vectors, countries as red triangles; supplementary variables are in capital letters.
